# Photo-induced energy-transfer polymerization

**DOI:** 10.1093/nsr/nwaf381

**Published:** 2025-09-10

**Authors:** Jian Liu, Yaxiong Wei, Liangwei Ma, Xin Jin, Siyu Sun, Shuyao Zhou, Xinsheng Xu, He Tian, Xiang Ma

**Affiliations:** Key Laboratory for Advanced Materials and Feringa Nobel Prize Scientist Joint Research Center, Frontiers Science Center for Materiobiology and Dynamic Chemistry, Institute of Fine Chemicals, School of Chemistry and Molecular Engineering, East China University of Science and Technology, Shanghai 200237, China; Anhui Province Key Laboratory of Optoelectric Materials Science and Technology, School of Physics and Electronic Information, Anhui Normal University, Wuhu 241002, China; Key Laboratory for Advanced Materials and Feringa Nobel Prize Scientist Joint Research Center, Frontiers Science Center for Materiobiology and Dynamic Chemistry, Institute of Fine Chemicals, School of Chemistry and Molecular Engineering, East China University of Science and Technology, Shanghai 200237, China; Key Laboratory for Advanced Materials and Feringa Nobel Prize Scientist Joint Research Center, Frontiers Science Center for Materiobiology and Dynamic Chemistry, Institute of Fine Chemicals, School of Chemistry and Molecular Engineering, East China University of Science and Technology, Shanghai 200237, China; Key Laboratory for Advanced Materials and Feringa Nobel Prize Scientist Joint Research Center, Frontiers Science Center for Materiobiology and Dynamic Chemistry, Institute of Fine Chemicals, School of Chemistry and Molecular Engineering, East China University of Science and Technology, Shanghai 200237, China; Department of Chemistry and Biochemistry, University of California, Los Angeles, CA 90095-1569, USA; Anhui Province Key Laboratory of Optoelectric Materials Science and Technology, School of Physics and Electronic Information, Anhui Normal University, Wuhu 241002, China; Key Laboratory for Advanced Materials and Feringa Nobel Prize Scientist Joint Research Center, Frontiers Science Center for Materiobiology and Dynamic Chemistry, Institute of Fine Chemicals, School of Chemistry and Molecular Engineering, East China University of Science and Technology, Shanghai 200237, China; Key Laboratory for Advanced Materials and Feringa Nobel Prize Scientist Joint Research Center, Frontiers Science Center for Materiobiology and Dynamic Chemistry, Institute of Fine Chemicals, School of Chemistry and Molecular Engineering, East China University of Science and Technology, Shanghai 200237, China

**Keywords:** polymerization, triplet–triplet energy transfer, photocatalyst

## Abstract

In conventional photo-induced polymerization strategies, the active species that initiate the reaction tend to be exogenous radical species. Inspired by photo-induced cycloaddition reactions, in this study, we investigated photo-induced polymerization from the perspective of energy-transfer processes. Utilizing low-energy, highly reactive triplet species of olefin molecules as energy acceptors, a polymerization strategy without the need for exogenous active components was developed. Triplet species from various sources were able to induce polymerization, demonstrating the excellent versatility of this strategy. The reaction mechanism was thoroughly investigated with controlled experiments and spectroscopic methods using thiochromanone as a template. It was clearly established that the key to polymerization is an active triplet species rather than a conventional radical species. As a result, the findings of this study stimulate further discussion on the role of monomers in photo-induced polymerization.

## Teaser

A polymerization reaction mechanism based on triplet energy transfer has been proposed, which exhibits a remarkably broad range of applications.

## INTRODUCTION

Photo-induced energy-transfer processes have been widely applied to the design of optical devices, solar cells and photocatalysts (PCs) [1,2]. For example, cycloaddition [3,4], bond dissociation [5,6] and transition metal sensitization [7,8] in coupling reactions have been carried out by using the photo-induced energy-transfer process. In these processes, a catalyst becomes photoexcited and undergoes triplet–triplet energy transfer (TTET) with the reactants, which in turn excites them to the active triplet state by crossing the energy gap. Among these reactions, the excitation wavelengths of many PCs have been extended to the visible region [2].

Acrylate and methacrylate molecules have been widely used in the preparation of polymer materials through various polymerization processes, but they are difficult to excite directly and often rely on the addition of exogenous active species [9–12]. It is also possible to achieve photopolymerization without traditional dyes or catalysts, but rather on the unique molecular structure of the monomers [13,14]. In reversible deactivation radical polymerization (RDRP)—an efficient method for the synthesis of polymers with more complex and well-defined structures—light is used as an initiator and regulatory switch for the reaction [15–17]. A series of organic PCs based on phenazine and phenothiazine were developed by Hawker and Miyake to achieve photo-controlled atom-transfer radical polymerization [18–21]. Photo energy-transfer reversible addition-fragmentation chain transfer (RAFT) polymerization was achieved through RAFT reagent engineering by Boyer *et al*. [22–24]. An abundance of photopolymerization methods can expand the range of applicable monomers [25–29] and optimize reaction conditions [30–32]. However, all these methods rely on the addition of exogenous radical species to achieve photopolymerization. Some cases involve more than three components, including a monomer, PC, initiator and modifier, thus complicating the system [33,34]. Moreover, while analyses of photo-induced polymerization frequently center on the photochemical processes involving catalysts, initiators and modifiers, the role played by the acrylate monomer in the reaction is commonly neglected. By revisiting photo-induced polymerization from the perspective of energy-transfer processes, the potential of acrylates and methyl acrylates to act as energy acceptors is revealed. This promotes our knowledge of polymerization reactions and assists in the design of polymerization methods with simpler components and more general applicability.

**Figure 1.**
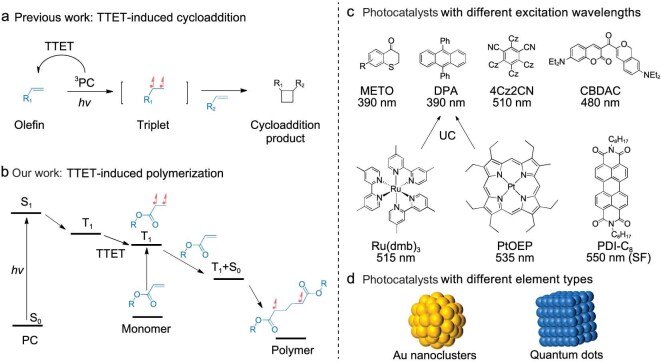
Process of photo-induced energy-transfer polymerization. Proposed mechanism of (a) TTET-induced cycloaddition reaction and (b) TTET-induced polymerization. (c, d) Various PCs mentioned in this article. UC, upconversion; SF, singlet fission; Cz, carbazole.

Herein, inspired by the mechanism of partial cycloaddition reactions (Fig. 1a) [4,35], it was realized that triplets of olefin molecules act as an active species in addition reactions. Moreover, acrylates are a special class of olefins that are often used as monomers in polymerization reactions. These facts aroused our interest in the polymerization of acrylates using triplet-state processes. This would differ from conventional radical polymerization, which is initiated by radicals generated by the photocleavage of organic molecules, and would be significantly distinguished from photo-RDRP, which controls the behavior of the active species through a photo-induced reversible reaction.

Acrylates commonly have very weak conjugated structures, which results in a high S_1_ energy level (>4.0 eV) and almost no absorption in the ultraviolet (UV)–visible region (Fig. S12). However, their double bonds rotate in the triplet state and this rotation causes energy dissipation, resulting in their T_1_ potential energy surface crossing with the S_0_ potential energy surface. This mechanism has been used to design photo-induced double-bond isomerization [36,37]. As a result, the T_1_ energy level of acrylates (∼2.6 eV) is much lower than the S_1_ energy level, which is difficult to reach through intersystem crossing (ISC). The lower T_1_ energy level also implies that acrylates are TTET acceptors for many photosensitizers. Therefore, photopolymerization may take place in more simplistic systems and allow the wavelength of the light source for photopolymerization to shift to as long a region as possible. The lifetimes of singlet states are generally <1/1000 of the triplet states and thus the contribution of the singlet states in the energy-transfer process is negligible. With this recognition, it is only the occurrence of the TTET process that is discussed in this paper.

## RESULTS

Based on the recognition of the TTET process, a two-component catalytic polymerization reaction consisting of merely the PC and the monomer was deemed attainable. During this process, the acrylate or methyl acrylate works as an acceptor that is excited to the triplet state by an energy-transfer process and becomes the initiation point of the chain reaction (Fig. 1b). Methoxyl-substituted thiochroman-4-one (METO), CBDAC and 4Cz2CN (Fig. 1d) were selected as TTET donors and their absorption wavelength distributions continuously covered the spectral range from 390 to 510 nm (Fig. S8).

The selected molecules with different excitation wavelengths all succeeded in catalysing the photo-induced polymerization of methyl methacrylate (MMA) (Table 1). The photo-induced polymerization by all the molecules was completed within 24 h and high final conversions of 92.2% and 90.9% were achieved by using METO-1 and 4Cz2CN, respectively. Triplet donor molecules with different structures and photochemical properties exhibited similar catalytic activity, undoubtedly demonstrating the universality and generality of the reaction.

**Table 1.** Polymerization using various photocatalysts.

	PC	Light (nm)	*E*_T1_ (eV)	Solvent	Conv (%)
1	METO-1	390	2.71	THF	92.2
2	CBDAC	480	2.34	THF	90.9
3	4Cz2CN	510	2.62	THF	81.3
4	AuNCs	365		H_2_O^a^	42.5^b^
5	CsPbBr_3_-OA	365		Hex	48.3^b^
6	CsPbBr_3_-RhB	365		Hex	51.2^b^
7	DPA	390	>2.88 (TTA)	DCM	55.6
8	Ru(dmb)_3_-DPA	510	>2.88 (TTA)	MeCN	32.4
9	Ru(dmb)_3_	510	2.02	MeCN	0
10	PtOEP-DPA	530	>2.88 (TTA)	DCM	27.0
11	PtOEP	530	1.90	DCM	0
12	CdSe-TCA-DPA	530	>2.88 (TTA)	Hex	26.4^b^
13	CdSe	530		Hex	0
14	PDI-C_8_	550	2.96 (SF)	Tol	68.2

Conversions were determined by using nuclear magnetic resonance (NMR). MMA as the monomer, reaction time = 24 h, PC loading = 500 ppm (relative to monomer), RT. (a) PEGMA_480_ as the monomer. (b) Conversions were determined by weight. PtOEP, platinum octaethylporphyrin. Ru(dmb)_3_: Tris(4,4'-dimethyl-2,2'-bipyridine) ruthenium(II). For more details, please refer to Table S5.

Under continuous light excitation, organic molecules may cleave and generate radicals that initiate polymerization reactions of monomers. Certain conventional radical initiators were designed based on this cleavage, such as azobisisobutyronitrile and dibenzoyl peroxide. As early as the 1980s, electron paramagnetic resonance (EPR) spectra were used to observe free-radical processes in the polymerization of acrylates and methyl acrylates [38–41]. Time-resolved electron paramagnetic resonance (TR-EPR) was able to show the generation and decay of these radicals [42]. In an effort to appreciate the completely innovative polymerization mechanism in this study, TR-EPR was used to capture radical species to exclude the effect of this process on the energy-transfer-induced polymerization reaction. TR-EPR experiments were undertaken for the METO-1 and METO-3 reaction systems before and after the addition of MMA, and no EPR peaks were detected (Figs S32 and S33) in the wider spectral range (330–340 mT). This indicated that no observable radicals were generated by either METO as a catalyst or the monomer during the reaction. Therefore, this experiment excluded the influence of radicals generated by the photocleavage of organic molecules on the reaction process in the system.

These energy-transfer processes are not exclusive to occurring between organic molecules and monomers. Some quantum dots (QDs) and Au nanoclusters (Au NCs) are favorable candidates for study, as they possess similar photochemical properties to those of organic molecules. The recently reported CsPbBr_3_ QDs with surface-anchored Rhodamine B enabled the efficient generation of triplet states by charge separation and the spin-flip mechanism [43]. This QD was also successfully applied to photo-induced polymerization, in which it demonstrated a tight correlation between the polymerization process and the energy-transfer process. To verify the findings, olein acid (OA)-modified CsPbBr_3_ QDs were also selected as a PC for the polymerization and similar results were obtained. Another inorganic catalyst—an Au@α-CD NC (Au NC) system stabilized in an aqueous phase—derives its properties from its nano-size effect. PEGMA_480_ was used as a monomer in photo-induced polymerization in an aqueous phase catalysed by Au NCs and the polymerization was also successful under similar yet different conditions. This suggests that energy-transfer-induced polymerization is broadly universal and does not depend on the catalyst type or solvent environment. The successful results convinced us of the existence of energy transfer and aroused our interest in its further study. The photochemical properties of these systems derive from a core consisting of inorganic elements, particularly OA-modified CsPbBr_3_ QDs and Au NCs, which are free of organic ligands with UV–visible absorption. Using these PCs excludes the possibility of generating free radicals from the cleavage of organic molecules under continuous irradiation. Their low conversion was due to the large proportion of acrylates in the reaction system (1:2 relative to the solvent, by volume), which increased the polarity of the solvent and caused some agglomeration of the QDs.

The only requirement for selecting a PC is an appropriate triplet-state energy level. With the energy condition satisfied, the polymerization can be induced by any triplets generated by various photophysical processes. Diphenylanthracene (DPA) is a common upconversion annihilator with fluorescence emission at ∼430 nm. A transition triplet species with a higher energy level than that of the singlet state arises during the triplet–triplet annihilation process [44–46], which generated a 55.6% polymerization conversion under direct excitation. Ru(dmb)_3_ and PtOEP acting as photosensitizers transferred their energy to DPA through an upconversion process under light excitations of 510 and 530 nm. The energy levels of their own triplet states are too low to induce polymerization, but, with the addition of DPA as an annihilating agent, polymerization occurred. Due to the limited efficiency of the upconversion process, the conversion rates of polymerization for these two approaches were only 32.4% and 27.0%. The triplet state of PDI-C_8_ has an energy level of ∼1.48 eV, which is not sufficient for TTET with monomer molecules, whereas a triplet state with double energy (2.96 eV) can be generated by singlet fission [47], which in turn induces a polymerization reaction.

**Figure 2.**
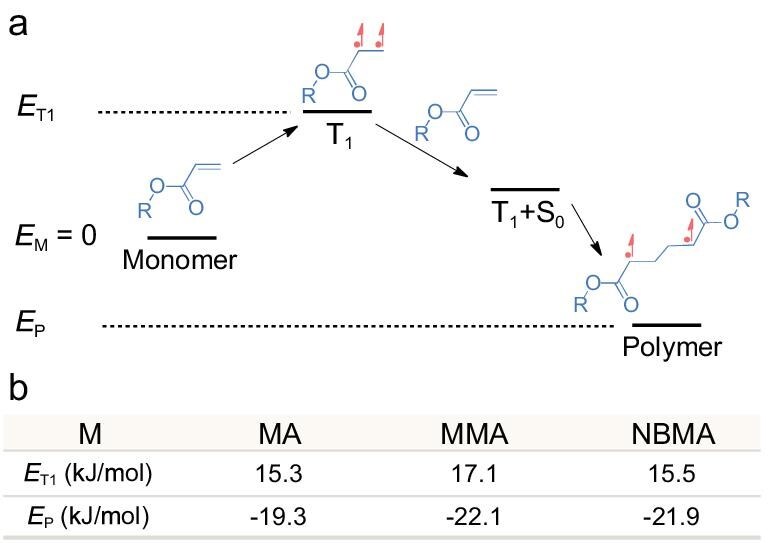
DFT results of photo-induced energy-transfer polymerization. (a) Scheme of photo-induced energy-transfer polymerization. (b) Energy gap between ground-state monomers, triplet-state monomers and polymer products in DFT calculations using different monomers.

The viability of this reaction in the field of thermodynamics has been explored in association with the aid of density functional theory (DFT) calculations. Figure 2a illustrates the starting point of the reaction, which is also an important primitive reaction in the polymerization process. The energy gaps given by DFT calculations show that, for several common monomers, the energy gaps between the monomer and polymer transition state are generally around −20 kJ/mol and the energy gaps for the triplet transition state and the polymer transition state converge to −35 kJ/mol. This demonstrates that the process is indeed feasible at room temperature, thereby providing a robust theoretical foundation for the theory.

To guide the design and selection of PCs for energy-transfer polymerization, the feasibility and mechanism of this energy-transfer phenomenon first needed to be investigated. According to the reaction mechanism proposed in Fig. 1b, the accumulation of triplet species is critical for initiating the polymerization reaction. Based on this presumption, the molar absorption coefficient, ISC efficiency and TTET efficiency are all considered important factors contributing to the generation and accumulation of triplet species.

**Figure 3.**
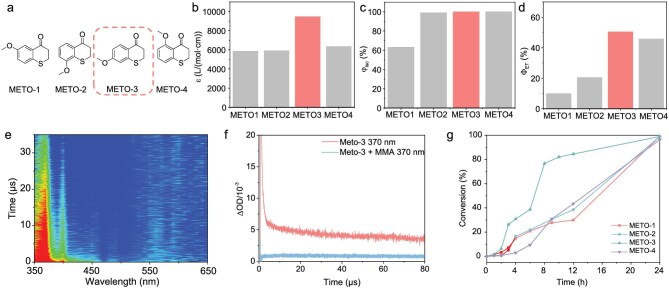
Spectrum data and catalytic performance of METOs. (a) Structures of METO-1–4. (b) Molar absorption coefficients of METO-1–4. (c) ISC efficiency of METO-1–4. (d) TTET efficiency between METO-1–4 and MMA. (e) Transition absorption (TA) spectra of METO-3. (f) Kinetic curve @370 nm of METO-3 before and after the addition of MMA. (g) Kinetic curves of the polymerization catalysed by METO-1–4.

Methoxyl-substituted thiochroman-4-one (METO) derivatives (Fig. 3a) are a class of molecules with unusual phosphorescent properties that were carefully studied by Ma *et al.* [48,49]. METO-1 to METO-4 molecules are isomers with different methoxy substituent positions, in which the molecular backbones are identical to single benzene structures. In addition to the varied position of the methoxy substituent, METO-1–4 can undergo significant changes in their molecular orbital distributions, which in turn change their k_f_ and thus greatly enhance their ISC efficiency for switching from fluorescence emission to phosphorescence.

To further elucidate the photochemical processes between METO molecules and MMA, we performed transient absorption experiments on the nanosecond scale (Figs S17–S29). As a class of molecules with millisecond lifetimes of phosphorescence emission at 77 K, the triplet species of METO are clearly and readily observed on transient absorption spectra. Consequently, such systems are suited for revealing their photochemical processes by comparing the positions of transient absorption peaks and kinetic decay curves in the presence and absence of MMA. For METO-3, the full-spectrum transient absorption quench after the addition of MMA was so pronounced that all transient absorption peaks within 300–800 nm were almost completely invisible (Fig. 3e). The kinetic curve of METO-3 showed a long-lived species of 50.7 μs at 370 nm and shorter lifetime species at 480 (0.42 μs) and 530 nm (0.64 μs). After the addition of MMA, there was no signal that could be fitted at 370 nm (Fig. 3f) or two other wavelengths. The quenching of the transient absorption spectra further confirmed the existence of the TTET process.

Combined with a comparison of the molar extinction coefficients, the ISC efficiencies and the energy transfer efficiencies (estimated by the quenching of the phosphorescent lifetimes), METO derivatives were predicted to be an efficient catalyst. METO-3 was considered to have the best catalytic performance (Fig. 3b–d) and the catalytic performances of METO-3 and METO-4 were better than those of METO-1 and METO-2. The kinetic curves of the photo-induced polymerizations were consistent with our expectations, with all four METOs achieving >90% catalytic conversion, while METO-3 had the highest conversion rate (Fig. 3g). Unfortunately, there were no transient absorbing species found for the energy-transfer product, probably due to the lower rate of generation and accumulation of the triplet species of MMA, making it difficult to accumulate detectable concentrations over the timescales tested.

Delicate experimental catalytic polymerization tests were carried out to visualize the effect of METO with different substituent positions on catalytic polymerization. The reaction proceeded readily at 100 ppm (relative to the monomer) and 500 ppm was sufficient for an almost complete conversion of MMA (Table 2). The photocatalytic performance of METO-1–4 was also consistent with the predictions. METO-1 had the lowest molar extinction coefficient, ISC rate and energy-transfer efficiency, and thus the lowest yield of the polymerization reaction. However, the molecular weight distributions were in the range of 1.3–1.5 under certain conditions, which was attributed to a lower reaction rate that eliminated several chain transfer and termination reactions. The catalytic performance of METO-3 and METO-4 was then regulated with a RAFT agent, which achieved narrow molecular weight distributions (1.1) and high initiation efficiencies (>90%) under suitable conditions (Table S10).

**Table 2.** Catalytic performance of METO-1–4.

PC	Loading (ppm)	*M*_n_ (kDa)	Đ	Conv (%)
METO-1	100	157.0	1.75	65.3
METO-1	500	68.3	1.61	92.2
METO-2	100	123.2	1.50	85.5
METO-2	500	75.4	1.57	>99
METO-3	100	542.8	1.30	95.6
METO-3	500	44.7	1.47	>99
METO-4	100	61.7	1.38	96.0
METO-4	500	65.7	1.58	>99

MMA as the monomer, THF as the solvent, reaction time = 24 h, light source = 390 nm, RT.

**Figure 4.**
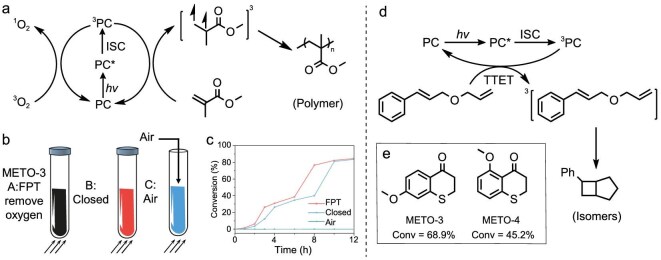
Experiments verifying the mechanism. (a) Scheme of TTET-induced photopolymerization. (b) Illustration of three different reaction conditions. (c) Polymerization rate profiles of METO-3 in three different oxygen environments. (d) Classical triplet-sensitized cycloaddition reaction. Reaction time = 6 h. (e) Conversion of METO-3 into METO-4 as a photocatalyst for the reaction in (d).

Aiming to demonstrate the causality between the energy-transfer process and the polymerization process, a comparative experiment of triplet-state quenching was introduced to provide evidence by observing the rates and conversions of the reaction. Oxygen is a specific molecule with a triplet state (^3^O_2_) as its ground state, enabling TTET with photosensitizers, and thus it is also a sensitive triplet-state quenching agent. The product of the TTET process is highly oxidizing singlet oxygen (^1^O_2_*, Fig. 4a) in which the excitation energy is as low as 0.98 eV (1265 nm) [50], which could not induce polymerization.

A radical process may also be inhibited by oxygen, yet we already excluded the effect of the radical process by TR-EPR in the previous discussion. Correspondingly, we also performed routine EPR testing. No significant EPR signals were observed in either the METO-1 or METO-3 samples (Figs S33–S36). As more direct evidence, we also characterized some molecules with smaller average molecular weights by using matrix-assisted laser desorption ionization time-of-flight mass spectrometry and did not observe any end groups with characteristic molecular weights, indicating that the initiating species in the system were not exogenous species, but the monomers themselves (Fig. S37).

Therefore, a control experiment was designed in which each reaction was carried out in parallel in three groups under different conditions. The deoxygenation conditions in the first group were controlled as three freeze–thaw cycles of deoxygenation and nitrogen filling. In the second group, the reaction system was closed to limit the oxygen content. In the third group, air was continuously passed in small volumes through an air pump to ensure fresh dissolved oxygen in the solution at all times (Fig. 4b). From the kinetic curves, it was evident that the rate of the second group using the closed system showed significant curve retardation in comparison with the rate curve of the first group, which indicated that there was a significant kinetic induction period in the reaction (Fig. 4c). In the first group, the freeze–thaw cycle produced deoxygenation, with almost no evidence of the TTET process between METO-3 and ^3^O_2_, and the production and accumulation of active species started immediately. In the second group, the quenching process of oxygen proceeded first and the accumulation of triplet reactive species began afterward. By 10–12 h, the conversions gradually tended to converge, which also indicated that the catalytic process is not a conventional radical-initiated process and the catalyst is quenched rather than consumed by the oxygen. There was only minimal polymerization because the pathways for the production and accumulation of active species were continuously inhibited by the continuous drumming of air in the third group. The same experiments were performed using METO-4 and an induction period was also observed (Fig. S30).

To achieve a closed-loop mechanism, we selected two well-studied energy-transfer-driven photochemical cycloaddition reactions [3], with acrylate derivatives and styrene derivatives as reactants and METO-3 and METO-4 as catalysts, and successfully achieved a certain conversion rate (Fig. 4d and Figs S42–S51), suggesting that there is an intrinsic correlation between the mechanisms of the two reactions. The fact that the reaction proceeded in two distinct pathways was due to differences in the structure and concentration of the reactants.

**Figure 5.**
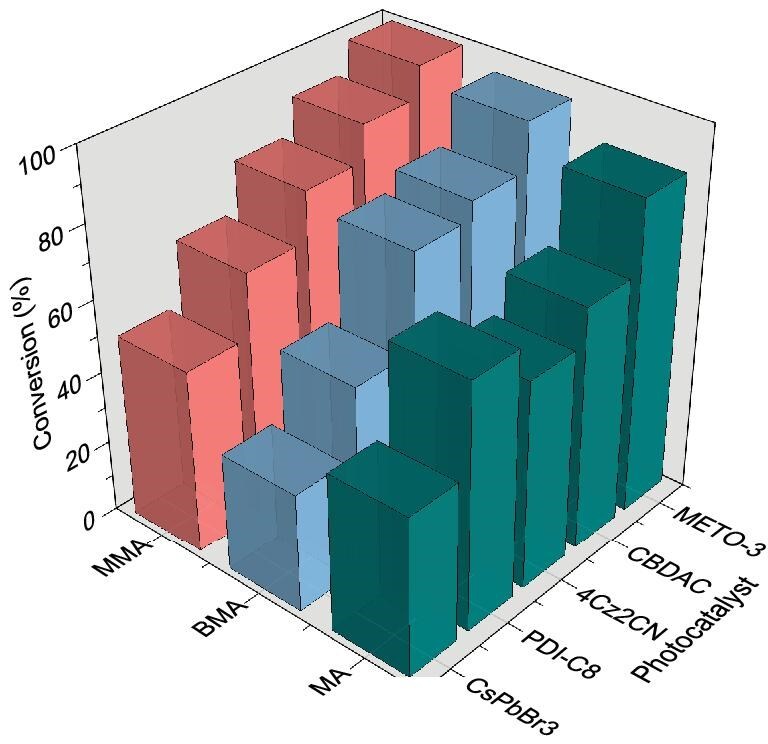
Catalytic performance of various photocatalysts with other acrylate monomers. PC loading = 500 ppm (relative to monomer), reaction time = 24 h, RT.

For ensuring the broad scope of this strategy, experiments of the catalytic polymerization of butyl methacrylate and methyl acrylate by several other selected PCs were also conducted (Fig. 5, details in Tables S6–S8). Within a larger range of monomers, the photocatalytic polymerization reaction remained capable of proceeding and the distribution of the yields achieved followed a similar trend to that exhibited by MMA.

## DISCUSSION

In conclusion, a novel photo-induced polymerization reaction pathway has been developed. The TTET process was successfully applied to excite monomer molecules to the triplet state as a starting point for chain reactions. According to the properties of the polymerization, the option of PCs is wide, including common organic molecules, as well as inorganic systems with similar photochemical properties. Photo-induced energy-transfer polymerization has less stringent requirements on the ISC efficiency of the PC and the small amount of triplet species generated by upconversion and singlet fission can meet the demand. The excellent universality of the TTET process simplifies the components of the polymerization system and certainly broadens the application potential of polymerization. The mechanism of the reaction was investigated and insightfully summarized by using transient spectroscopy and TR-EPR was used to exclude interference from conventional radical polymerization. Based on the postulated reaction mechanism, PCs were designed and selected, and the expected properties were obtained. The results of TR-EPR confirm the existence of this process as distinguished from conventional radical polymerization.

## METHODS

Any methods, additional references and source data are available in the Supplementary information.

## Supplementary Material

nwaf381_Supplemental_File

## Data Availability

All relevant data that support the findings are available within this article and supporting information, and are also available from authors upon reasonable request.
