# Supplementary material for: Photo-induced energy-transfer polymerization
Source: Natl Sci Rev. 2025 Sep 10;12(12):nwaf381. doi: 10.1093/nsr/nwaf381 (PMC12684163; doi:10.1093/nsr/nwaf381)
Supplement: nwaf381_Supplemental_File [file nwaf381_supplemental_file.pdf]

Supplementary Information for

Photo-induced Energy Transfer Polymerization

Jian Liu[1], Yaxiong Wei[2], Liangwei Ma[1], Xin Jin[1], Siyu Sun[1], Shuyao Zhou[3], Xinsheng Xu[2], He Tian[1], Xiang Ma[1,]*

Author Information

[1] Key Laboratory for Advanced Materials and Feringa Nobel Prize Scientist Joint Research Center, Frontiers Science Center for Materiobiology and Dynamic Chemistry, Institute of Fine Chemicals, School of Chemistry and Molecular Engineering, East China University of Science and Technology, Meilong Road 130, Shanghai 200237, P. R. China

[2]Anhui Province Key Laboratory of Optoelectric Materials Science and Technology, School of Physics and Electronic Information, Anhui Normal University, Wuhu 241002, China.

[3]Department of Chemistry and Biochemistry, University of California, Los Angeles, CA 90095-1569, USA.

The PDF file includes:

Materials and Methods
Supplementary Text
Figs. S1 to S46
Tables S1 to S11
References

Supplementary Information for

Photo-induced Energy Transfer Polymerization

Materials and Methods **3**
Materials 3
General Instruments 3
Supplementary Text **4**
General polymerization procedure: 4
Synthesis: 5
Basic spectral data of photocatalysts and monomers: 11
Basic spectral data of METOs: 15
Transient spectral data of photocatalysts before and after the addition of MMA: 19
Polymerization reaction details: 25
TR-EPR spectra of METO-1 and METO-3: 27
^{1}H NMR spectra of polymers and method for determining conversion: 30
Photocatalysis of another well-studied triplet-sensitized photochemical reaction: 33
Light source data mentioned in this article: 40
References 43

Materials and Methods

Materials: Methyl methacrylate (MMA, 99.9%), methyl acrylate (MA, 99.9%), butyl methacrylate (BMA, 99.9%), poly(ethylene oxide) methyl ether methacrylate (PEGMA, 99%) and 3-Bromopropionic acid were purchased from Shanghai Adamas Reagent. p-Methoxybenzenethiol, o-Methoxybenzenethiolm m-Methoxybenzenethiol, 2,3,5,6-Tetra(9H-carbazol-9-yl)terephthalonitrile (4Cz2CN, 97%), 2,9-Dioctylanthra[2,1,9-def:6,5,10-d'e'f']diisoquinoline-1,3,8,10(2H,9H)-tetraone (PDI-C_8, 95%), 3,3'-Carbonylbis(7-(diethylamino)-2H-chromen-2-one) (CBDAC, 95%), Platinum octaethylporphyrin (PtOEP, 95%) and 4-Cyano-4-(dodecyl-sulfanylthiocarbonyl)sulfanylpentanoic acid (CTA, 95%) were purchased from Shanghai Bide Reagent. Cadmium oxide (CdO, 99.999%), oleic acid (OA, 99%), dodecanethiol (DDT, 98%), 1-octadecene (ODE, 90%), were purchased from Sigma-Aldrich. Selenium (Se, 99.999%) and tri-n-octylamine (TOA, 95%) was purchased from Aladdin. Ethanol and hexane were purchased from DAMAO. The monomer (MMA) is purified by decompression distillation and stored at -20 ℃ for up to two weeks.

General Instruments: ^{1}H NMR spectra were measured on a Brüker AV400 spectrometer. The UV-vis absorption spectra and PL spectra were performed on a Varian Cary 500 spectrophotometer at 25 ℃.

Transient Absorption (TA) Measurements. Nanosecond time-resolved transient absorption spectra and decay kinetics were obtained using an LFP instrument (LP 980, Edinburgh Instruments Ltd). The pump laser beam and the probe beam crossed perpendicularly through the liquid sample in a quartz cuvette (10 mm × 10 mm). Samples were excited by 355 nm laser pulses (10 Hz, 7.0 mJ per pulse) which were delivered by the third harmonic of a Nd:YAG laser (Surelite II-10, Continuum Inc.). A dynamic decay curve was recorded with a digital phosphor oscilloscope (TDS 3012C, Tektronix Inc.). The samples were placed in a sealed quartz cuvette and performed three FPT cycle to remove oxygen.

Time-resolved electron paramagnetic resonance (TR-EPR) Measurements. Chemically induced dynamic electron polarization (CIDEP) spectra were recorded on an X-band EPR spectrometer (JES-X310, JEOL RESONANCE Inc.) without magnetic field modulation. The hardware of this instrument mainly consists of a microwave unit, a cavity resonator, a spectrometer and an electromagnet. An aqueous sample cell with an optical path of 0.3 mm enables high-sensitivity CIDEP measurements. A high-speed photodiode detector (Si photodiode, Hamamatsu Photonics) with a high sensitivity of 0.38 A • W^{-1} and a low dark current (max.) of 50 pA is suitable for synchronous triggering of an oscilloscope with laser pulse. PBQ shows strong absorption at 355 nm, and the sample inside the resonator was excited by the 355 nm laser pulses (10 Hz, 4.5 mJ per pulse, pulsewidth ≈ 8 ns) which were delivered by the third harmonic of a Nd: YAG laser (Surelite II-10, Continuum Inc.). A constant flow pump (HL-2S, Shanghai Huxi Analysis Instrument Factory Co., Ltd.) was used to circulate the sample solution through the aqueous sample cell with an output flow speed of 500 mL h^{-1} to avoid the temperature increase in the local position of the cell. The microwave unit adopts the detection method of homodyne-reflection diode rectification.

Size Exclusion Chromatography (SEC): SEC measurements were performed on an Agilent 1260 Infinity II System, comprising PLgel MIXED-D, 7.5 × 300 mm, 5 μm, HPLC column, and a differential refractive index (RI) detector using THF as eluent at 35 ℃ with a flow

rate of 1 mL·min^{-1}. The SEC system is calibrated using linear poly styrene standards (PL2010-0301 EasiVial PS-M, 2 mL, pre-weighed calibration kit).

Triplet-triplet annihilation photon upconversion (TTA-UC)

Preparation of the TTA system. The CdSe@CdS QD-9-anthracene carboxylicacid (ACA) complexes were prepared by adding ACA powders into the QD solution in hexane, followed by sonication and filtration. Then, QD-ACA complexes were mixed with diphenyl anthracene (DPA) in hexane. After degassed in glovebox, the mixture was sealed in custom-made airtight cuvettes for optical measurements.

TTA-UC measurements. We used a 589 nm *cw* laser as the laser source. The excitation path was along the sidewall of the cell and the PL was collected perpendicularly, which was focused into a fiber-coupled spectrometer (Maya2000 Pro, Ocean Optics). The upconversion quantum yield (Φ_{UC}') was calculated using coumarin 503 (C503) as a reference with a quantum yield of 0.75 in ethanol. The Φ_{UC}' (normalized to 100%) was calculated according to the following equation:

$$\Phi'_{UC} = 2\Phi_{C503}\frac{1-10^{-A_{C503}}}{1-10^{-A_S}}\cdot\frac{F_S}{F_{C503}}\cdot\frac{{n_S}^2}{{n_{C503}}^2} \quad \text{(equation 1)},$$

where "*S*" and "*C503*" represent the QD-ACA//DPA complexes and the reference C503, respectively. *Φ*, *A*, *F*, and *n* are the photoluminescence quantum yield, the absorbance at the excitation wavelength, the integrated emission photon number, and the solvent refractive index used for measurement, respectively.

Supplementary Text

General polymerization procedure: Reaction condition: The polymerization was conducted with MMA (2.12 mL, 10.0 mmol, 100 eq.) as the model monomer, added to solvents containing PC, the amount of solvent was adjusted so that the volume of the whole system was 4 mL (6 mL when the solvent is DMSO). Add the mixture to the quartz tube with a PTFE stirring bar. The mixture was deoxygenated by three freeze–pump–thaw cycles, filled with argon and sealed up. And then the polymerization was occurred under irradiation by a light source sheet of the specified wavelength, at room temperature. The cooling water was fed into the external circulation system of the photoreactor casing to keep the system temperature below 30 ℃. After 24 hours of polymerization, the casing pipe was opened and 30 μL of the mixture was syringed out and quenched into $CDCl_3$ containing 250 ppm BHT to determine the monomer conversion by ^{1}H NMR. Diluted the mixture with 1 mL of DCM to reduce its viscosity, then dripped into 50 ml methanol and stirred for more than 2 hours. Through vacuum filtration the product was collected again and dried under reduced pressure to obtain a white powder.

Polymerization procedure of the evaluate of kinetic curve of different PC (Table 2):

Reaction condition: The polymerization was conducted with MMA (3.18 mL, 15.0 mmol, 100 eq.) as the monomer, added to solvents containing PC, the amount of DCM was adjusted so that the volume of the whole system was 6 mL. Add the mixture to the quartz tube with a PTFE stirring bar. The mixture was deoxygenated by three freeze–pump–thaw cycles, filled with argon and sealed up. And then the polymerization was occurred under irradiation by a light source sheet of the specified wavelength, at room temperature. The cooling water was fed into the external circulation system of the photoreactor casing to keep the system temperature below 30°C. After a while, use a syringe to puncture the sealing plug to draw 30 μL of the mixture and quenched into $CDCl_3$ containing 250 ppm BHT to determine the monomer conversion by ^1H NMR, then press the sealing plug tightly to keep the system airtight. Repeat this process to get conversions at multiple points in time.

Polymerization procedure of the evaluate of kinetic curve of different condition (Figure 4): Reaction condition: The polymerization was conducted with MMA (4.4 mL, 60 mmol, 100 eq.) as the monomer, added to solvents containing PC, the amount of DMSO was adjusted so that the volume of the whole system was 10 mL. Add the mixture to the quartz tube with a PTFE stirring bar. And then the polymerization was occurred under irradiation by a light source sheet of the specified wavelength, at room temperature. The cooling water was fed into the external circulation system of the photoreactor casing to keep the system temperature below 30°C. After a period of time, use a syringe to puncture the sealing plug to draw 30 μL of the mixture and quenched into $CDCl_3$ containing 250 ppm BHT to determine the monomer conversion by ^1H NMR, then press the sealing plug tightly to keep the system airtight.

Synthesis: The thiochromanone molecules METO-1 to METO-4, Au@CD NCs and all of quantum dots mentioned were synthesized according to our previous work.[1-3]

The cycloaddition reaction used as a template (Figure 4d) for the control study was carried out exactly according to the conditions in reference [4]. A 390 nm light source was applied, and the reaction time was 6 h.

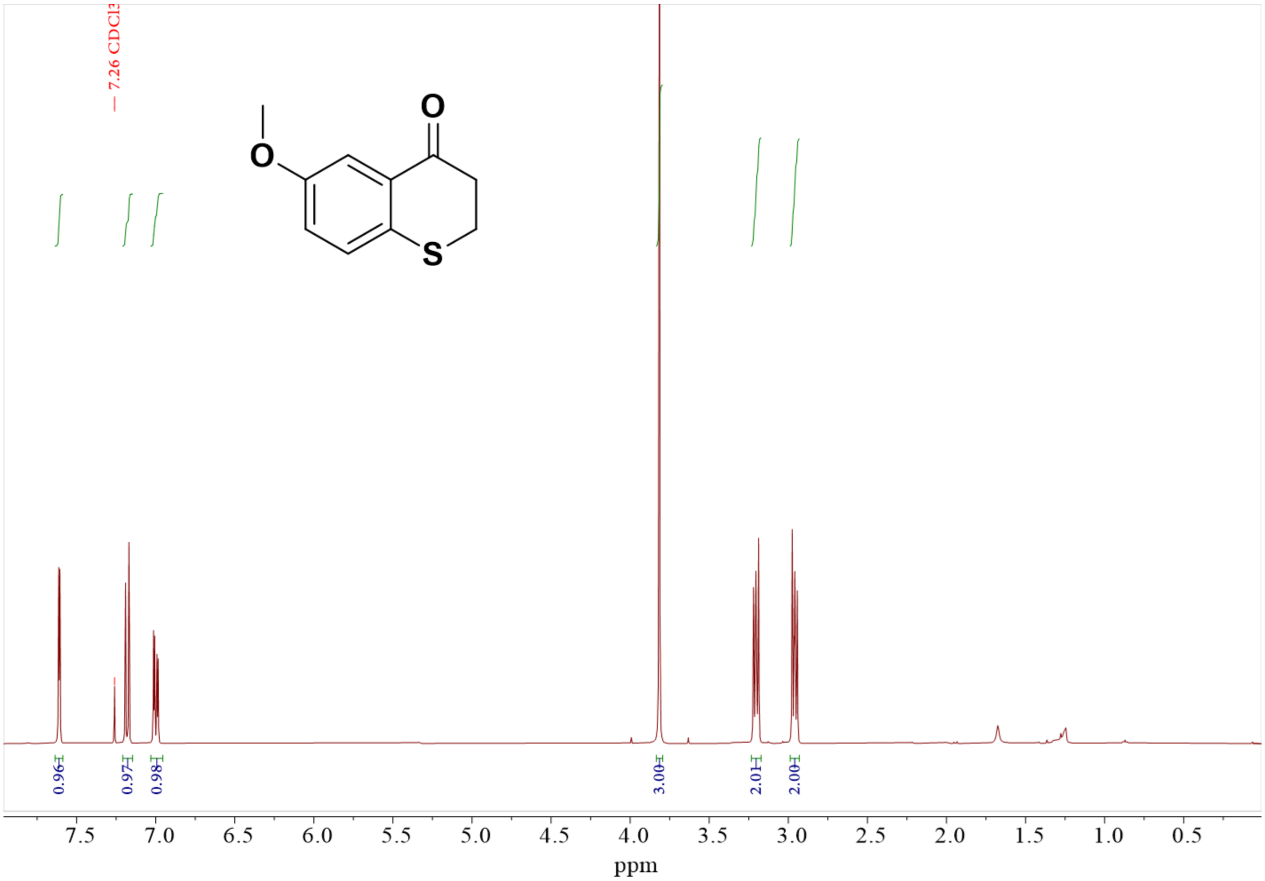

Figure S1. ^{1}**H NMR spectrum of METO-1. (CDCl$_3$)**

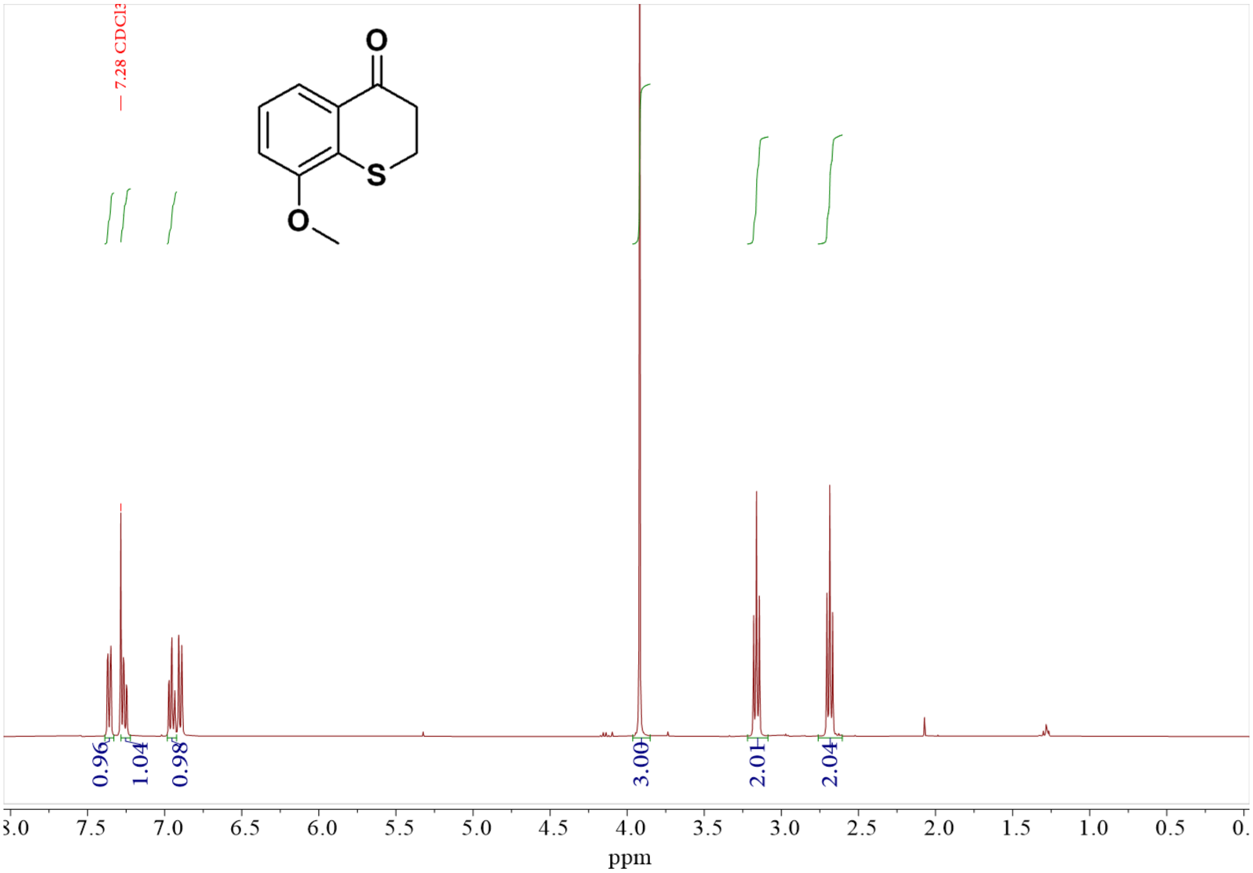

Figure S2. ^{1}**H NMR spectrum of METO-2. ($CDCl_3$)**

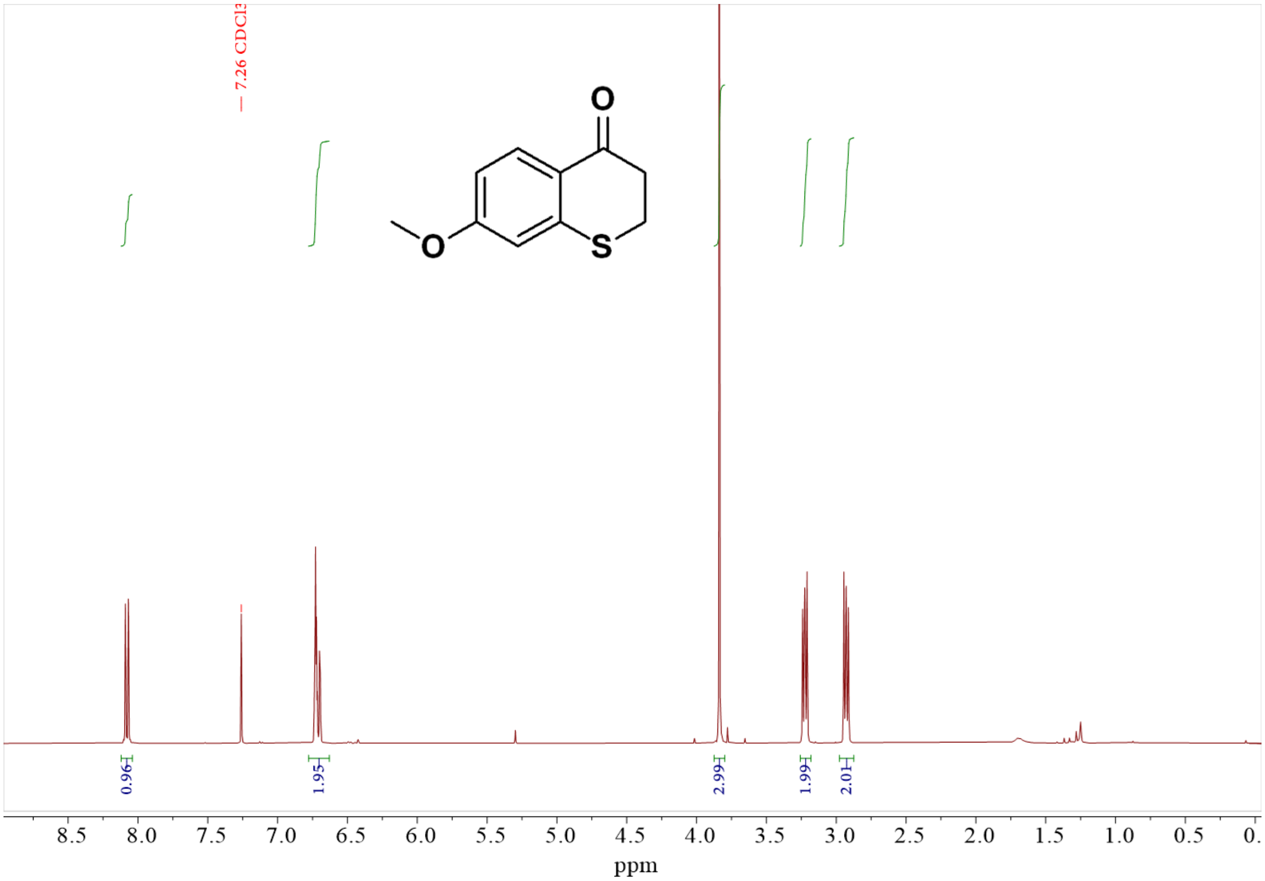

Figure S3.^{1}H NMR spectrum of METO-3. ($CDCl_3$)

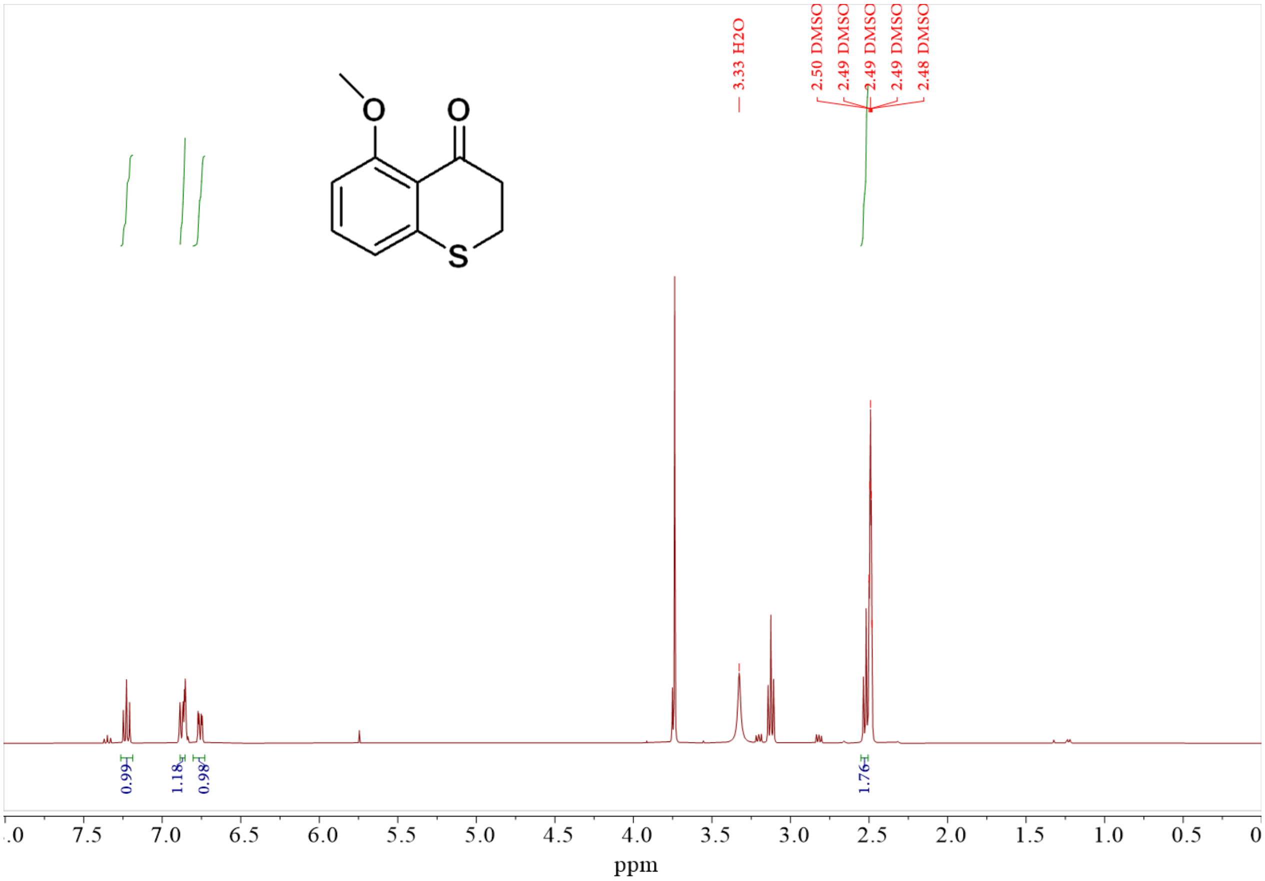

Figure S4. ^{1}H **NMR spectrum of METO-4. (DMSO-D$_6$)**

Synthesis of CdSe and CdSe/CdS QDs

The CdSe core QDs are synthesized using a reported method [5]. 0.5 M Cd-oleate stock solution was prepared first. Typically, 10 mmol CdO, 10 ml OA, and 10 ml ODE was loaded in a three-neck flask and degassed at 100 °C for 10 min. Then, the mixture was saturated with N_2 and heated to 280 °C. The reaction was maintained at 280 °C for 20 min then cooled to room temperature. The obtained 0.5 M Cd-oleate solution was kept at 100 °C for further use. In order to synthesize CdSe cores, 1 ml of 0.5 M Cd-oleate and 10 ml ODE was loaded in a three-neck flask and degassed at 100 °C in vacuum for 10 min. Then, the reaction was filled with N_2 and heated to 260 °C. We inject 2.5 ml of 0.1 M Se-ODE suspension (prepared by dispersing 1 mmol Se powder in 10 ml ODE) into the reaction then react at 250 °C for 8 min. Then we added the Se-ODE suspension dropwise into the reaction and monitored the absorption of the CdSe QDs by taking the aliquots. When the first exciton absorption peak of CdSe reached 520 nm, the reaction

was stopped and cooled to room temperature. The obtained CdSe cores were purified with ethanol and hexane and dispersed in 5 ml hexane for further use.

The CdSe/CdS QDs with band-edge exciton absorption peak at 545 nm were synthesized through a literature method with minor modification [6]. We mixed 1 ml of obtained CdSe cores, 1 ml of 0.5 M Cd-oleate solution, and 10 ml TOA in a three-neck flask and degassed at 100 ^{o}C for 10 min. Then, we filled the reaction with N_2 and heated it to 300 ^{o}C. When the temperature reached 300 ^{o}C, we added a desired amount of DDT dropwise into the reaction and monitored the absorption of the CdSe/CdS QDs by taking the aliquots. When the first exciton absorption peak of CdSe/CdS reached 545 nm, we stopped the reaction and purified the CdSe/CdS QDs with ethanol and hexane. The CdSe/CdS QDs were stored in 5 ml hexane for further use.

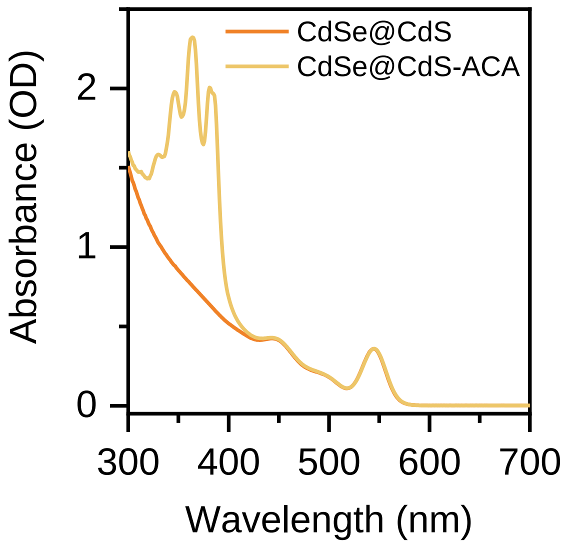

Figure S5. Absorption spectra of CdSe@CdS and CdSe@CdS-ACA.

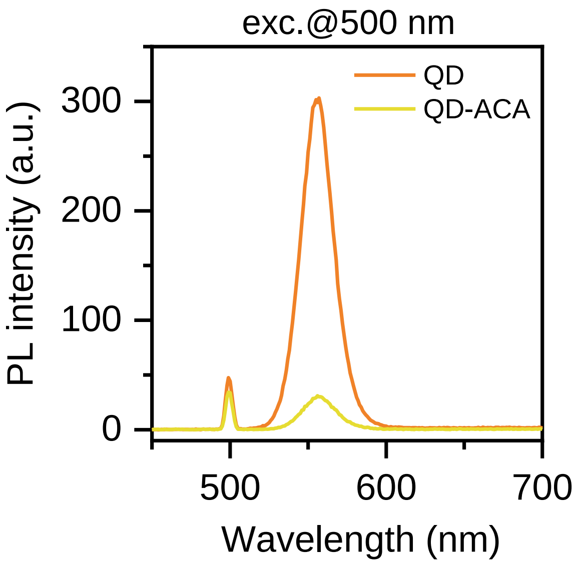

Figure S6. Emission spectra of CdSe@CdS and CdSe@CdS-ACA. Excitation wavelength = 500 nm.

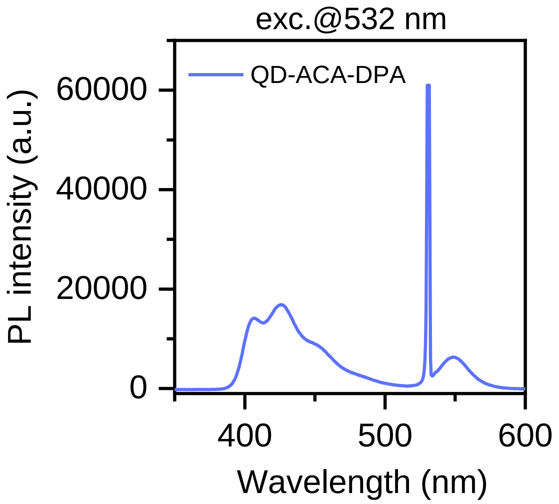

Figure S7. Upconversion fluorescent emission spectra of CdSe@CdS-ACA-DPA. Excitation wavelength = 532 nm.

Basic spectral data of photocatalysts and monomers:

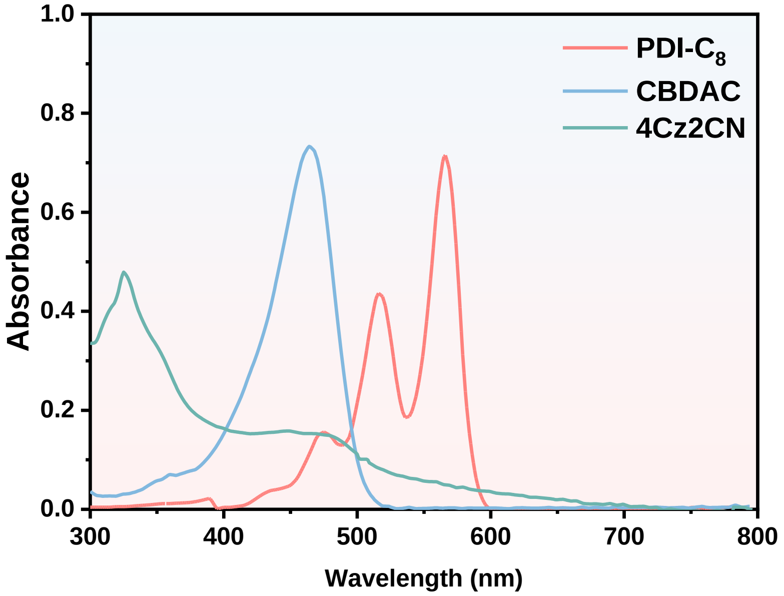

Figure S8. Absorption spectra of long wavelength-PC mentioned by this article. Solvent = Toluene (PDI-C_8), DCM (CBDAC), DCM(4Cz2CN). Concentration = 0.1 mM (PDI-C_8), 0.05Mm(CBDAC), 0.1mM (4Cz2CN).

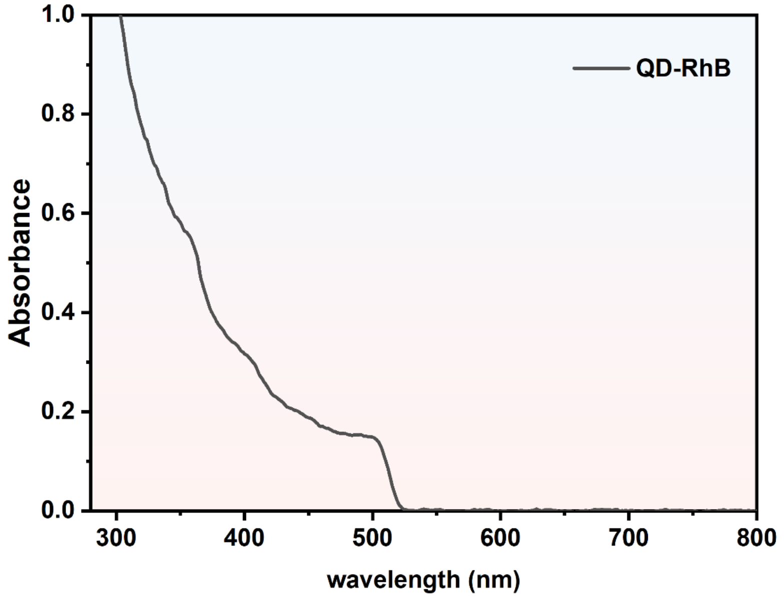

Figure S9. Absorption spectra of $CsPbBr_3$-RhB.

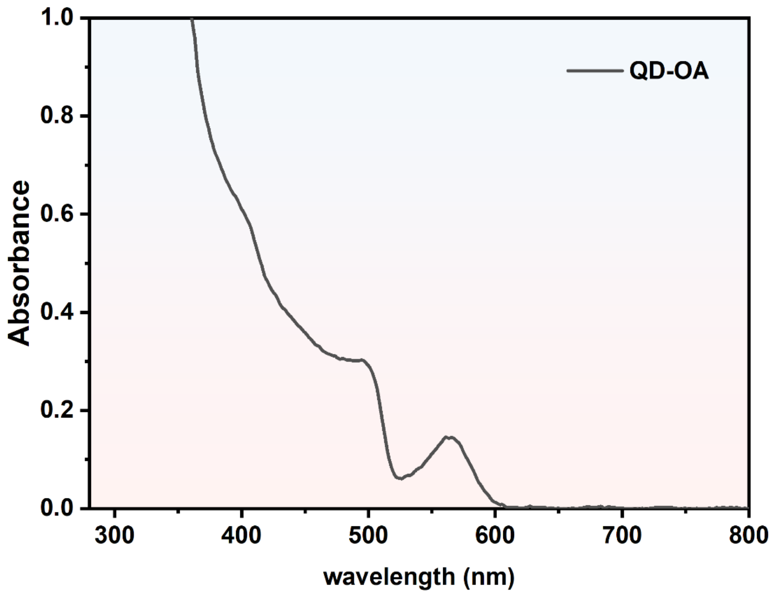

Figure S10. Absorption spectra of $CsPbBr_3$-OA.

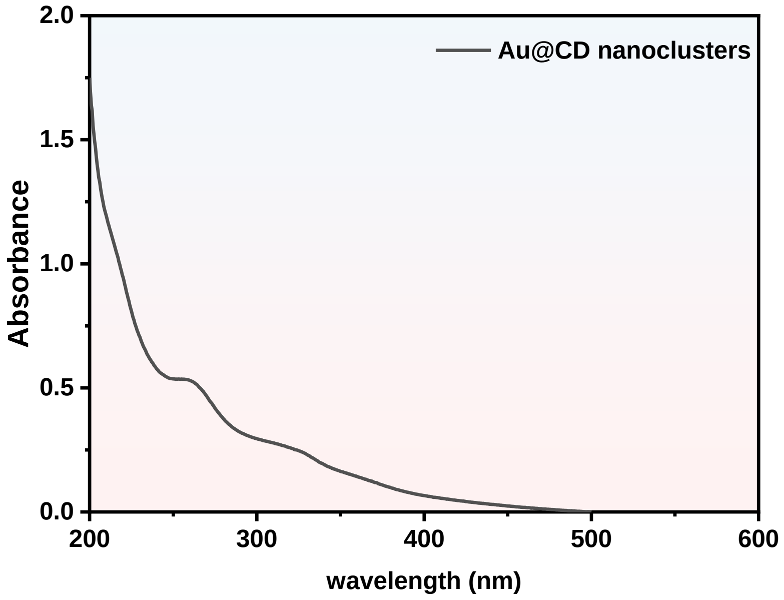

Figure S11. Absorption spectra of Au@α-CD nanoclusters.

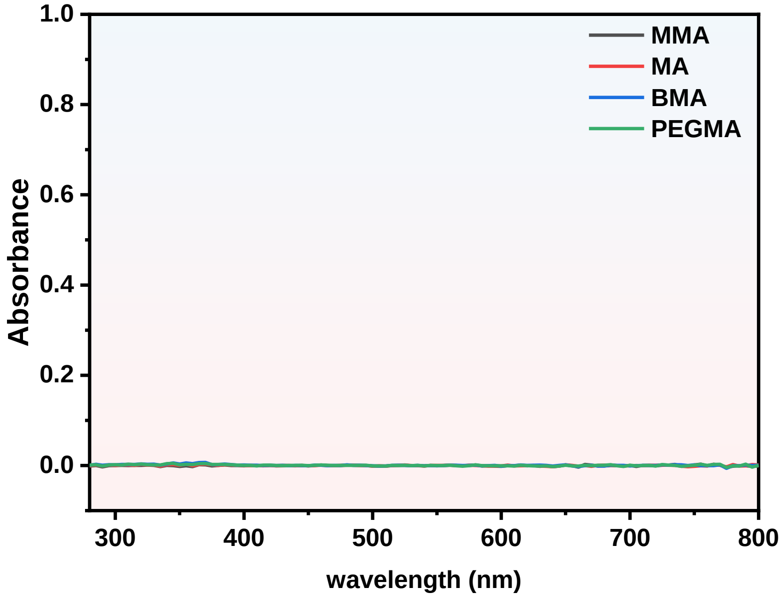

Figure S12. Absorption spectra of all monomers mentioned by this article.

Basic spectral data of METOs:

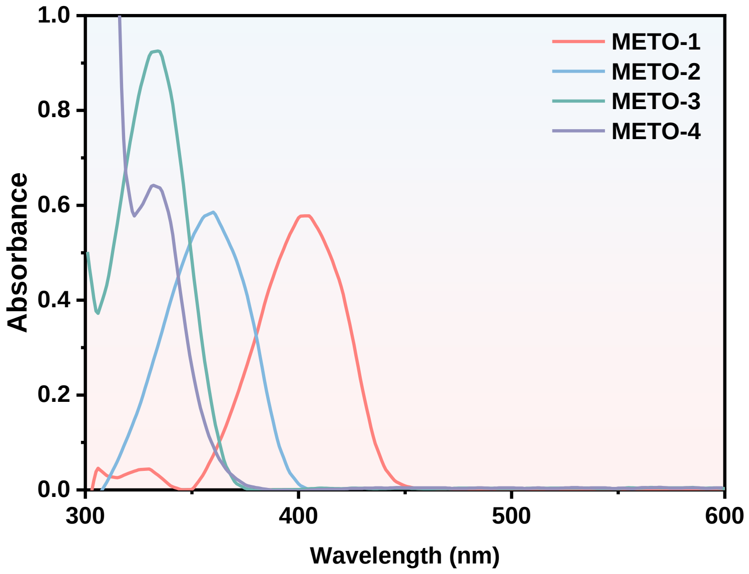

Figure S13. Absorption spectra of METO-1~4. Solvent = THF.

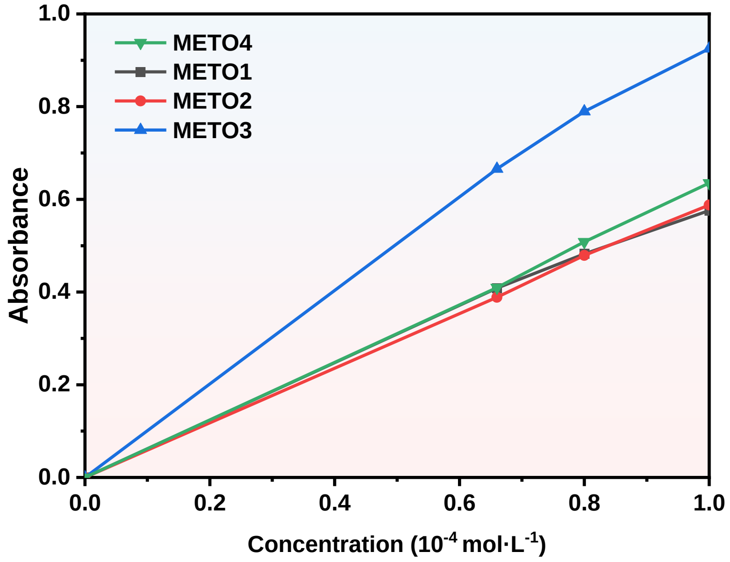

Figure S14. Determination by absorption spectra of molar extinction coefficient of METO-1~4.

Table S1. Molar extinction coefficient of METO-1~4.

	**METO-1**	**METO-2**	**METO-3**	**METO-4**
ε (L/(mol cm))	5851	5907	9457	6344

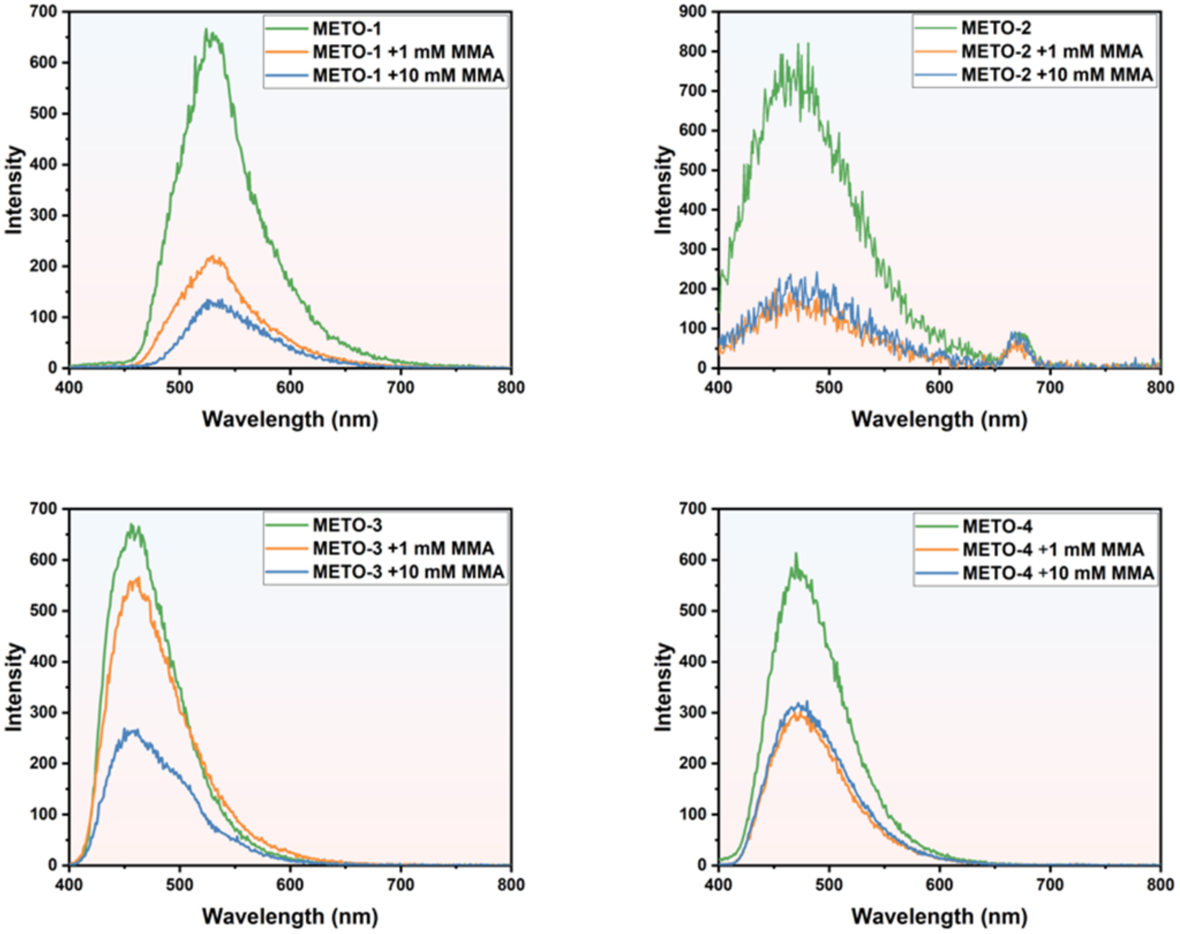

Figure S15. Phosphorescence emission spectra of METO-1~METO-4 under the presence of different concentration of MMA. Green line: METO-1~4. Orange line: under the presence of 1 mM MMA. Blue line: under the presence of 10 mM MMA. c(METO) = 10^{-4} mol • L^{-1}, λ_{ex} = 360 nm, solvent = DMSO, measured under 77 K.

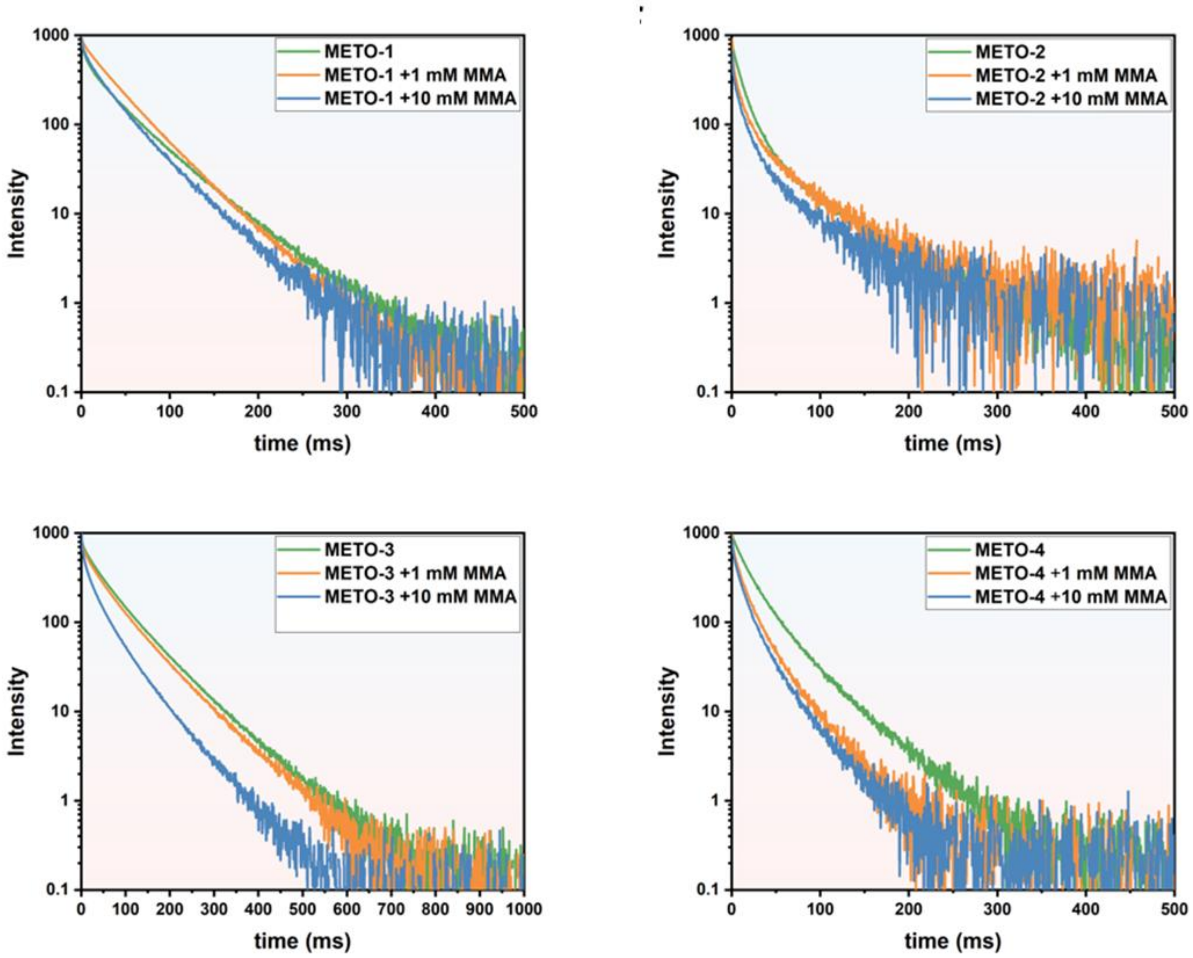

Figure S16. Lifetime spectra of phosphorescence emission of METO-1~METO-4. Green line: METO-1~4. Orange line: under the presence of 1 mM MMA. Blue line: under the presence of 10 mM MMA. c(METO) = 10^{-4} mol • L^{-1}, λ_{ex} = 390 nm, solvent = DMSO, measured under 77 K.

Table S2. The phosphorescence lifetime of METO-1~METO-4 under different concentration of MMA. c(METO) = 10^{-4} mol • L^{-1}, λ_{ex} = 390 nm, solvent = DMSO, measured under 77 K.

Donor	τ_p (ms)	τ_p (ms) (c(MMA) = 10 mM)	τ_p (ms) (c(MMA) = 10 mM)
METO1	32.2	36.1	29.0
METO2	13.2	10.6	10.5
METO3	60.0	55.4	29.8
METO4	23.4	14.3	12.7

$$\Phi_{ET} = 1 - \frac{\tau_{DA}}{\tau_D}$$

Φ_{ET}: Energy-transfer efficiency. τ_{DA}: The phosphorescence lifetime of METO-1~METO-4 under the presence of MMA.τ_D: The phosphorescence lifetime of METO-1~METO-4.

Table S3. Energy-transfer efficiency between METO-1~4 and MMA.

	METO-1	METO-2	METO-3	METO-4
Φ_{ET}(%)	9.94	20.45	50.33	45.73

Transient spectral data of photocatalysts before and after the addition of MMA:

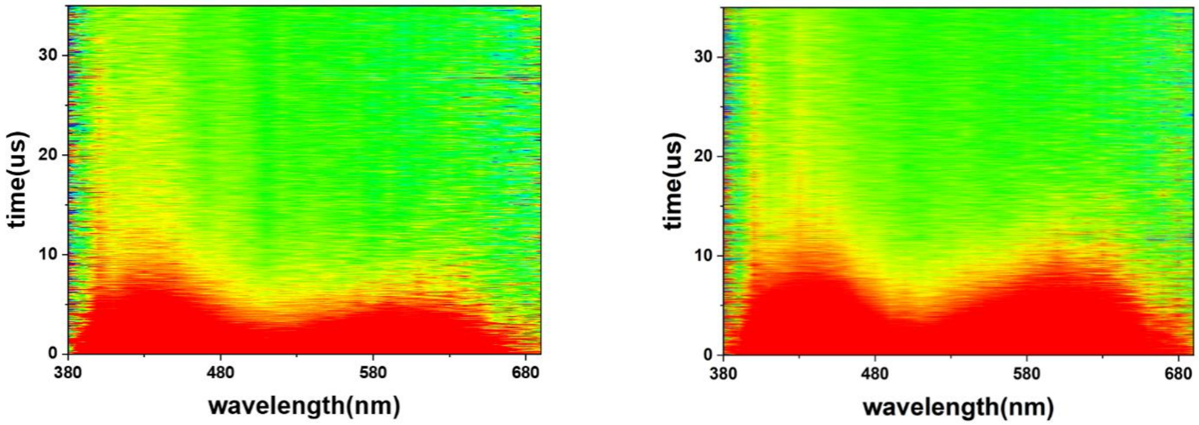

Figure S17. Transient absorption (TA) spectra of METO-1 at full spectrum (left). Transient absorption (TA) spectra of METO-1 and MMA at full spectrum (right).

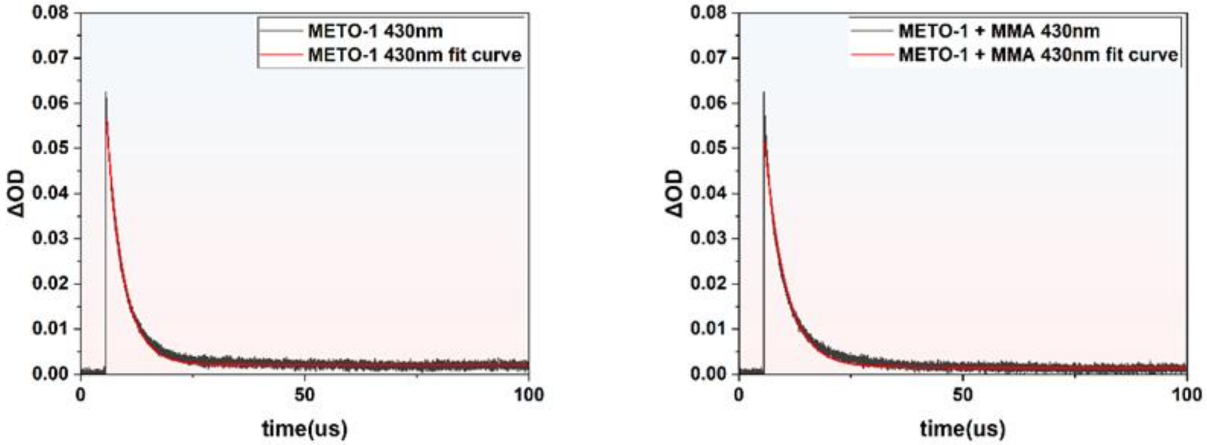

Figure S18. Transient kinetic decay curves for METO-1 at 430 nm (left). Transient kinetic decay curves for METO-1 and MMA at 430 nm (right).

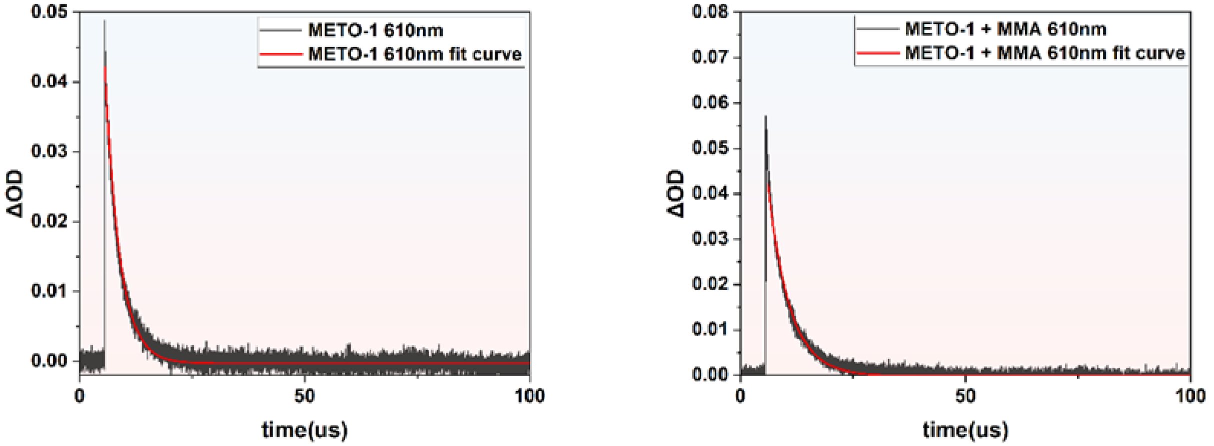

Figure S19. Transient kinetic decay curves for METO-1 at 610 nm (left). Transient kinetic decay curves for METO-1 and MMA at 610 nm (right).

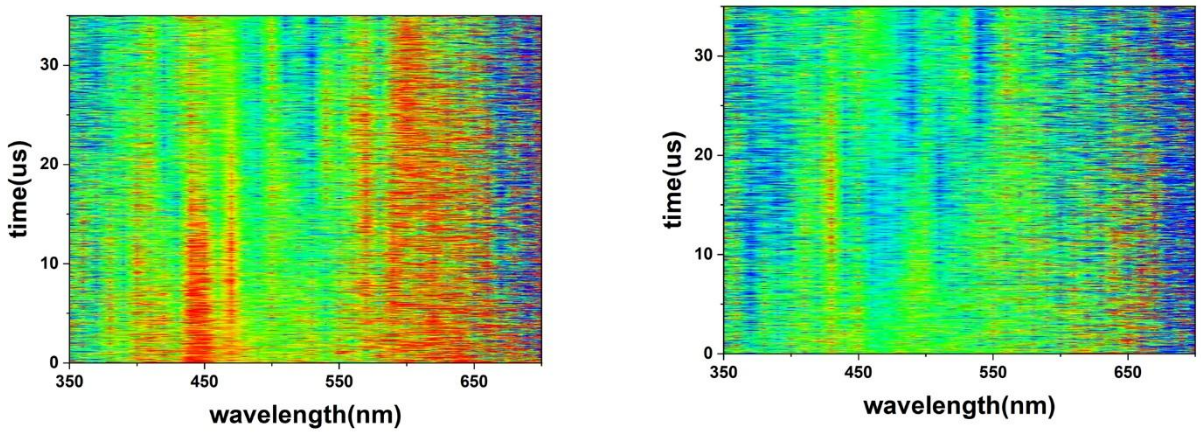

Figure S20. Transient absorption (TA) spectra of METO-2 at full spectrum (left). Transient absorption (TA) spectra of METO-2 and MMA at full spectrum (right).

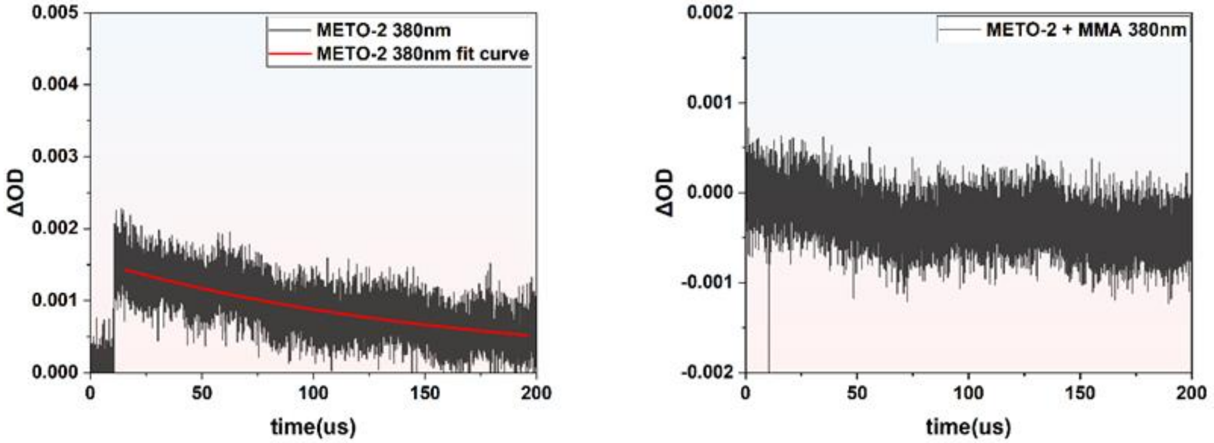

Figure S21. Transient kinetic decay curves for METO-2 at 380 nm (left). Transient kinetic decay curves for METO-2 and MMA at 380 nm (right).

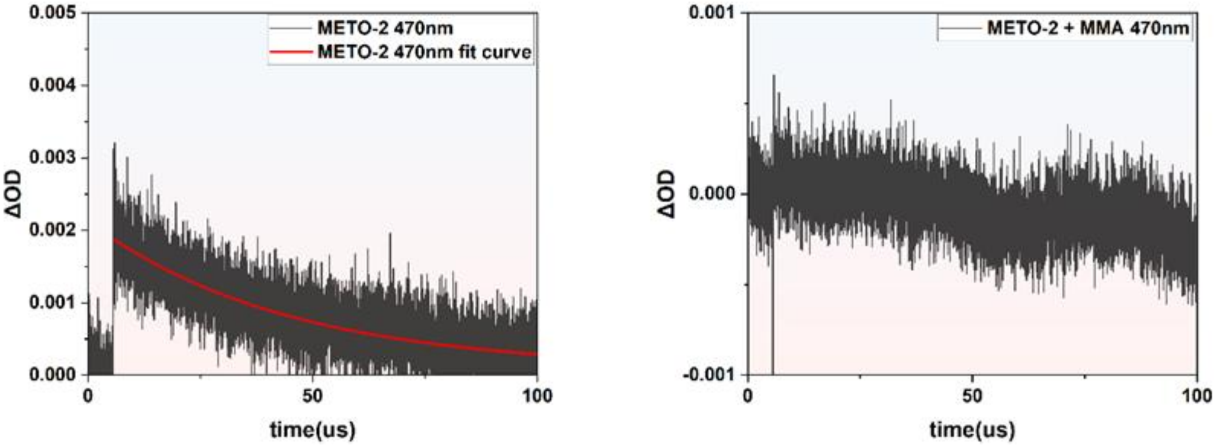

Figure S22. Transient kinetic decay curves for METO-2 at 470 nm (left). Transient kinetic decay curves for METO-2 and MMA at 470 nm (right).

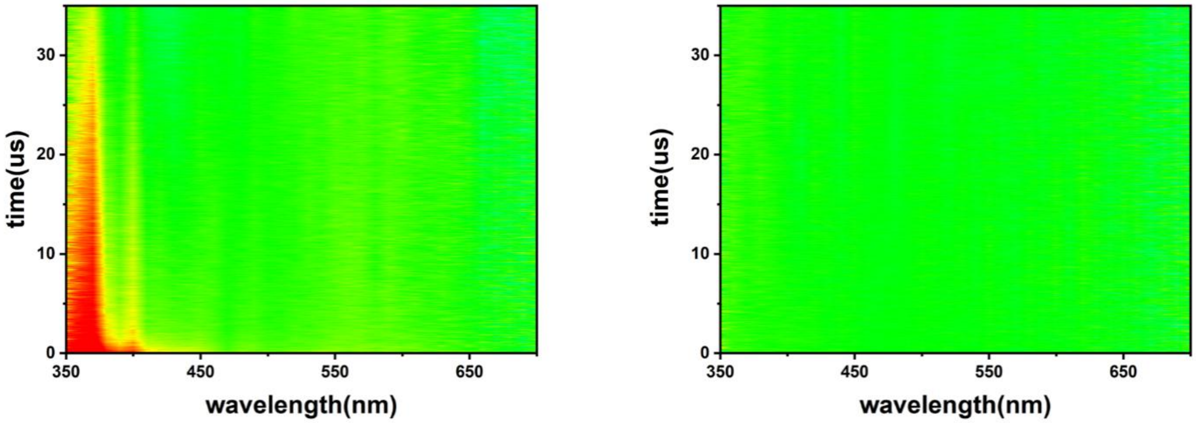

Figure S23. Transient absorption (TA) spectra of METO-3 at full spectrum (left). Transient absorption (TA) spectra of METO-3 and MMA at full spectrum (right).

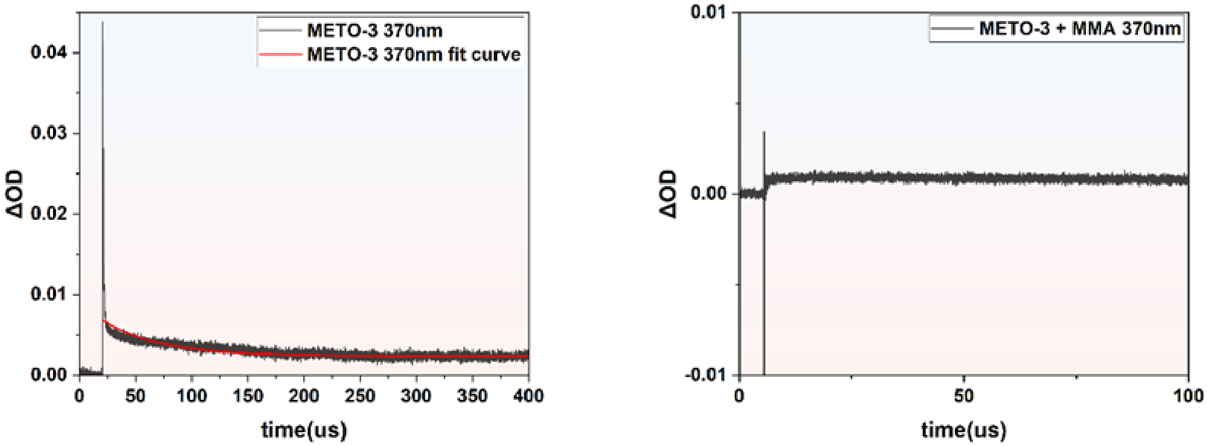

Figure S24. Transient kinetic decay curves for METO-3 at 370 nm (left). Transient kinetic decay curves for METO-3 and MMA at 370 nm (right).

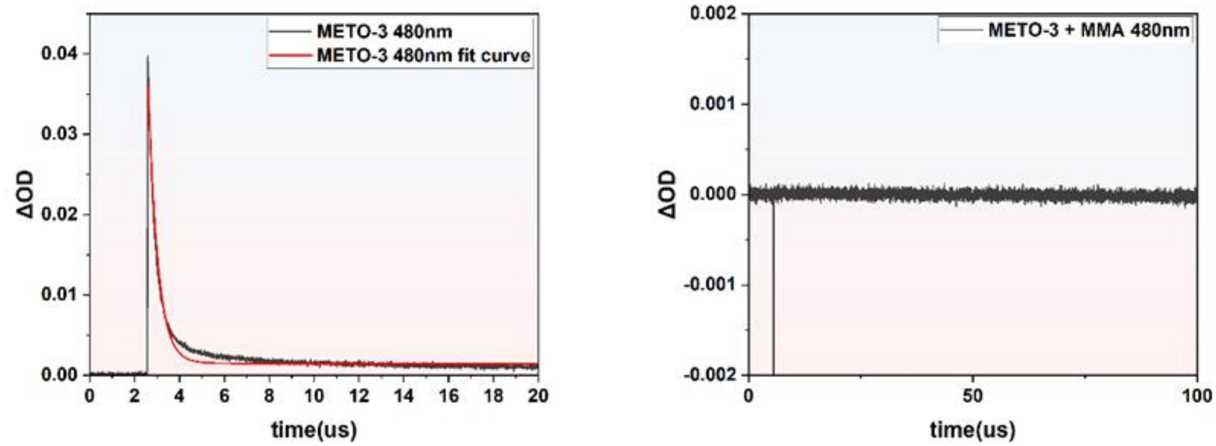

Figure S25. **Transient kinetic decay curves for METO-3 at 480 nm (left). Transient kinetic decay curves for METO-3 and MMA at 480 nm (right).**

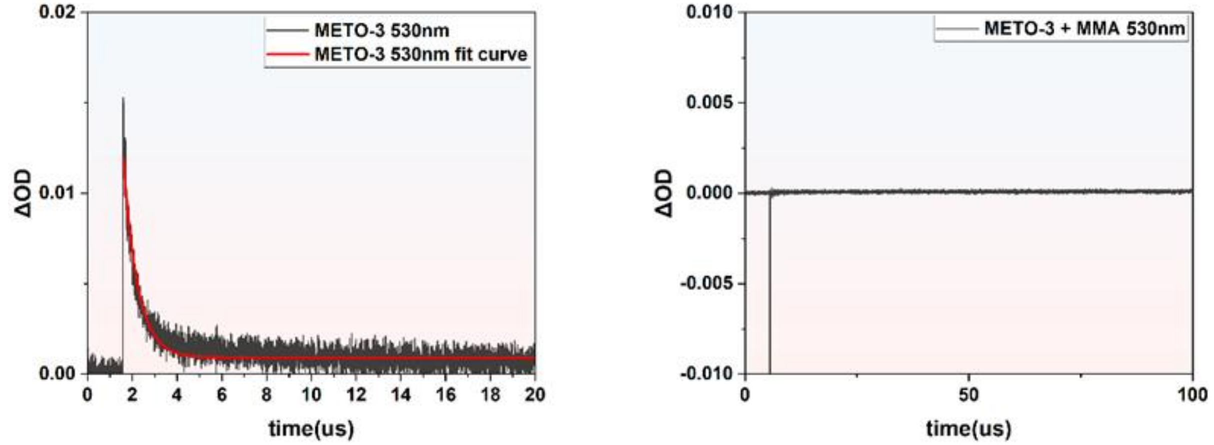

Figure S26. **Transient kinetic decay curves for METO-3 at 530 nm (left). Transient kinetic decay curves for METO-3 and MMA at 530 nm (right).**

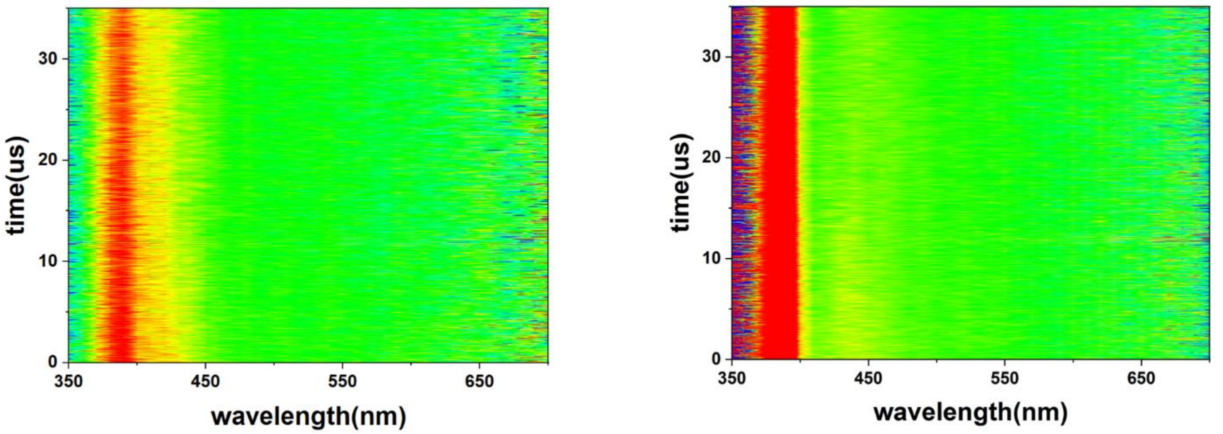

Figure S27. Transient absorption (TA) spectra of METO-4 at full spectrum (left). Transient absorption (TA) spectra of METO-4 and MMA at full spectrum (right).

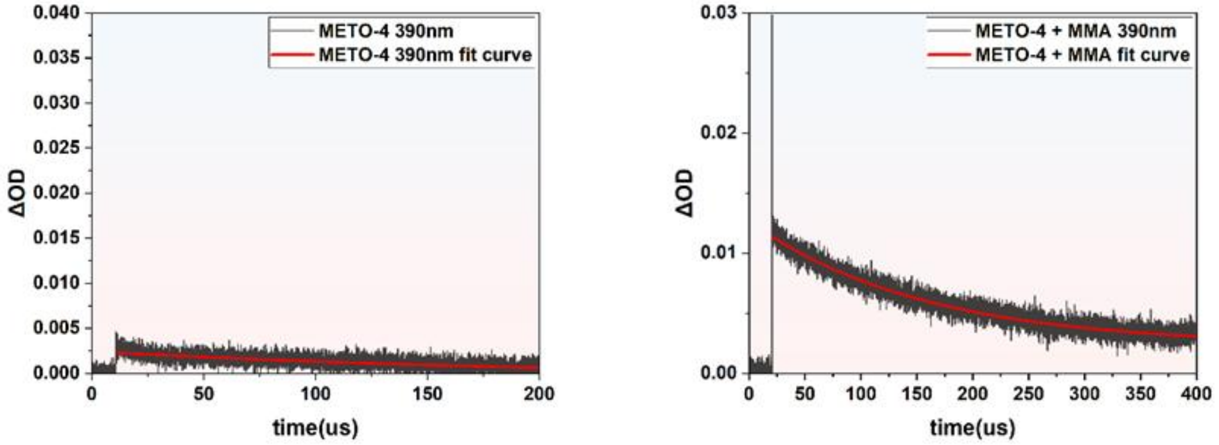

Figure S28. Transient kinetic decay curves for METO-4 at 390 nm (left). Transient kinetic decay curves for METO-4 and MMA at 390 nm (right).

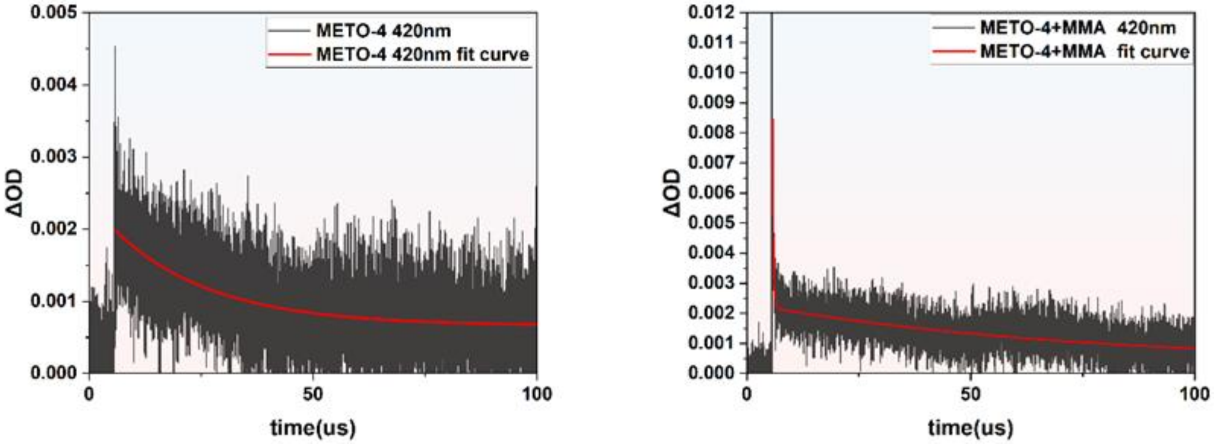

Figure S29. Transient kinetic decay curves for METO-4 at 420 nm (left). Transient kinetic decay curves for METO-4 and MMA at 420 nm (right).

Table S4. Lifetime of transient species, fitted by transient kinetic decay curves.

**METO-1**	**λ= 430 nm**	**λ= 610 nm**	
τ_a (us)	5.19	3.26	
τ_b (us)	4.64	4.47	
**METO-2**	**λ= 380 nm**	**λ= 470 nm**	
τ_a (us)	160	43.7	
τ_b (us)	/	/	
**METO-3**	**λ= 370 nm**	**λ= 480 nm**	**λ= 530 nm**
τ_a (us)	50.7	0.424	0.636
τ_b (us)	/	/	/
**METO-4**	**λ= 390 nm**	**λ= 420 nm**	
τ_a (us)	213	21.8	
τ_b (us)	153	21.7	

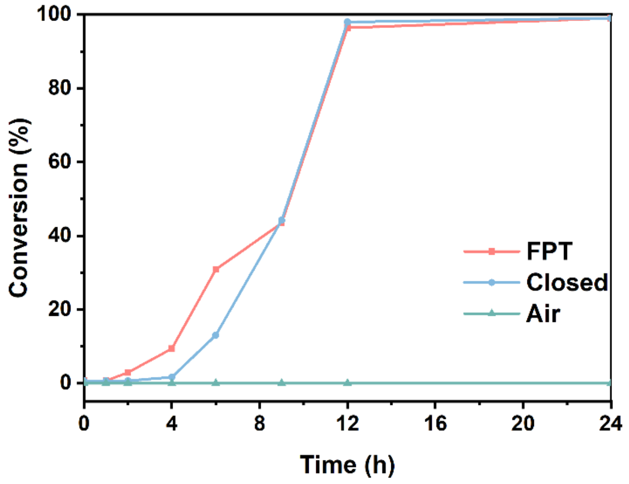

Figure S30. Polymerization rate profiles of METO-4 in three different oxygen environments. Reaction process is the same as METO-3.

Polymerization reaction details:

Table S5. Catalyst performance of other PC. MMA as the monomer, reaction time = 24 h, RT.

PC	light	solvent	Mn(kDa)	Mw(kDa)	Đ	Conv (%)
METO-1	390	THF	68.3	110.0	1.61	92.2
CBDAC	480	THF	76.2	115.5	1.52	90.9
4Cz2CN	510	THF	138.6	241.3	1.74	81.3
AuNCs	365	H_2O	7.4	9.0	1.22	42.5[b]
$CsPbBr_3$-OA	365	Hex	263.8	377.9	1.43	48.3[b]
$CsPbBr_3$-RhB	365	Hex	93.3	162.5	1.74	59.8[b]
DPA	390	DCM	102.2	171.0	1.67	55.6
DPA	530	DCM	/	/	/	/
$Ru(dmb)_3$-DPA	510	MeCN	84.2	122.3	1.45	32.4
PtOEP-DPA	530	DCM	64.9	99.4	1.53	27.0
CdSe-TCA-DPA	530	Hex	80.2	123.2	1.54	26.4[b]
PDI-C_8	550	Tol	381.0	517.5	1.36	68.2
$Ru(dmb)_3$	510	MeCN	/	/	/	/
PtOEP	530	DCM	/	/	/	/
CdSe	530	Hex	/	/	/	/

Table S6. Catalyst performance of other PC. BMA as the monomer, reaction time = 24 h, RT.

PC	light	solvent	Mn(kDa)	Mw(kDa)	Đ	Conv (%)
METO-3	390	THF	25.9	35.8	1.38	94.9
CBDAC	480	THF	135.7	234.8	1.73	82.3
4Cz2CN	510	THF	115.5	185.0	1.60	77.7
PDI-C8	550	Toluene	63.1	110.1	1.75	51.7
QD-RhB	550	Hexane	107.6	199.0	1.85	33.2

Table S7. Catalyst performance of other PC. MA as the monomer, reaction time = 24 h, RT.

PC	light	solvent	Mn(kDa)	Mw(kDa)	Đ	Conv (%)
METO-3	390	THF	41.3	60.6	1.46	86.6
CBDAC	480	THF	32.5	47.9	1.47	67.4
4Cz2CN	510	THF	56.4	81.4	1.44	57.9
PDI-C8	550	Toluene	40.3	59.2	1.47	68.2
QD-RhB	550	Hexane	48.7	69.1	1.42	44.2

Table S8. Catalyst performance of other PC. St as the monomer, reaction time = 24 h, RT.

PC	light	solvent	Mn(kDa)	Mw(kDa)	Đ	Conv (%)
METO-3	390	THF	26.2	40.5	1.55	58.3
CBDAC	480	THF	24.3	35.0	1.44	57.5
4Cz2CN	510	THF	15.6	24.9	1.72	47.9
PDI-C8	550	Toluene	2.4	3.3	1.40	35.4

Table S9. Catalyst performance of METO-1~METO-4. MMA as the monomer, Light sourse = 390 nm, reaction time = 24 h, RT.

PC	Loading	solvent	Mn(kDa)	Mw(kDa)	Đ	Conv (%)
METO1	100	THF	157.0	274.0	1.75	65.3
METO1	500	THF	68.3	110.0	1.61	92.2
METO2	100	THF	123.2	184.4	1.50	85.5
METO2	500	THF	75.4	118.5	1.57	>99
METO3	100	THF	542.8	706.1	1.30	95.6
METO3	500	THF	44.7	65.6	1.47	>99
METO4	100	THF	61.7	85.4	1.38	96.0
METO4	500	THF	65.7	103.7	1.58	>99

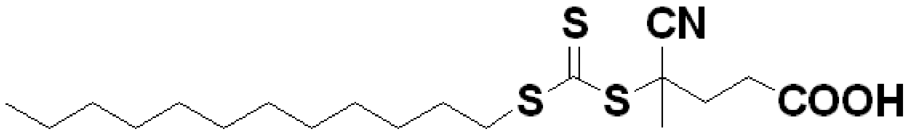

Figure S31. Structure of CTA.

Table S10. RAFT catalyst performance of METO-3~METO-4. I: initiate efficiency. MMA as the monomer, Light sourse = 390 nm, reaction time = 24 h, RT. M_m, molecular weight of monomer, α, conversion.*Conduct under the dark.

PC	CTA%	Mn(kDa)	Mw(kDa)	Đ	Conv (%)	I(%)
METO3	1	10.1	11.6	1.15	92.5	92
METO3	0.1	134	167	1.25	88.3	66
METO3	0.01	960	1060	1.10	83.3	87
METO4	1	7.90	9.40	1.19	80.2	102
METO4	0.1	126.9	168	1.32	74.5	59
METO4	0.01	736	855	1.16	69.2	94
/	1	/	/	/	/	/
METO3*	1	/	/	/	/	/

$$CTA\% = \frac{n(monomer)}{n(CTA)} * 100 \qquad I(\%) = \frac{M_m * \alpha}{M_n * CTA\%}$$

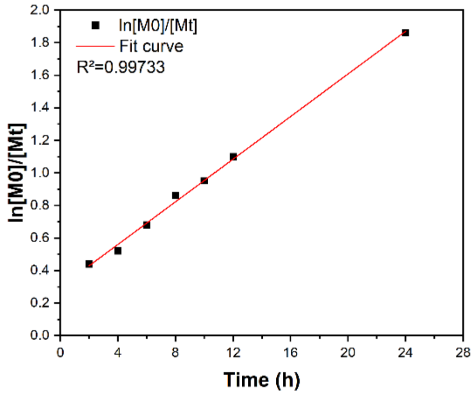

Figure S32. Kinetic monitoring of the reaction after adding RAFT reagent. MMA as the monomer, Light sourse = 390 nm, PC= METO-3 (500ppm).

TR-EPR spectra of METO-1 and METO-3:

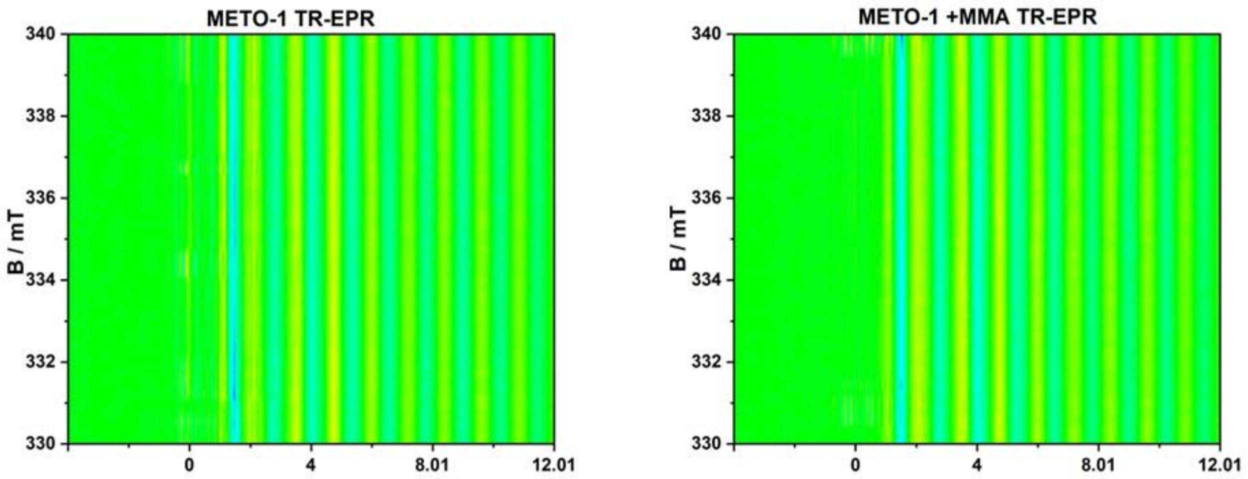

Figure S33. TR-EPR spectra of METO-1 and METO-1+MMA. c(METO) = 10^{-3} mol • L^{-1}, c(MMA) = 10^{-2} mol • L^{-1}. λ_{ex} = 355 nm, solvent = isopropanol.

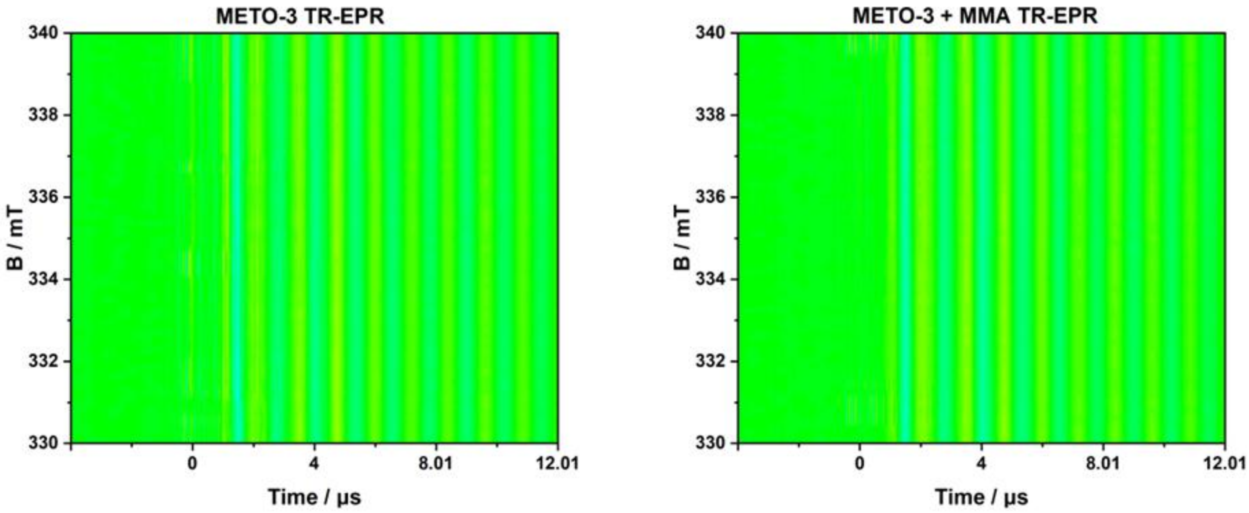

Figure S34. TR-EPR spectra of METO-3 and METO-3+MMA. c(METO) = 10^{-3} mol • L^{-1}, c(MMA) = 10^{-2} mol • L^{-1}. λ_{ex} = 355 nm, solvent = isopropanol.

Correspondingly, we also performed routine EPR testing. First, METO-1 and METO-3 (194 mg) were added to 1 mL of THF (containing 0.5 wt% DMPO), followed by 0.1 mL of MMA. After bubbling to remove oxygen, the mixture was exposed to a 390 nm light source for 30 minutes, and EPR testing was then performed rapidly. No significant EPR signals were observed in either

the METO-1 or METO-3 samples.

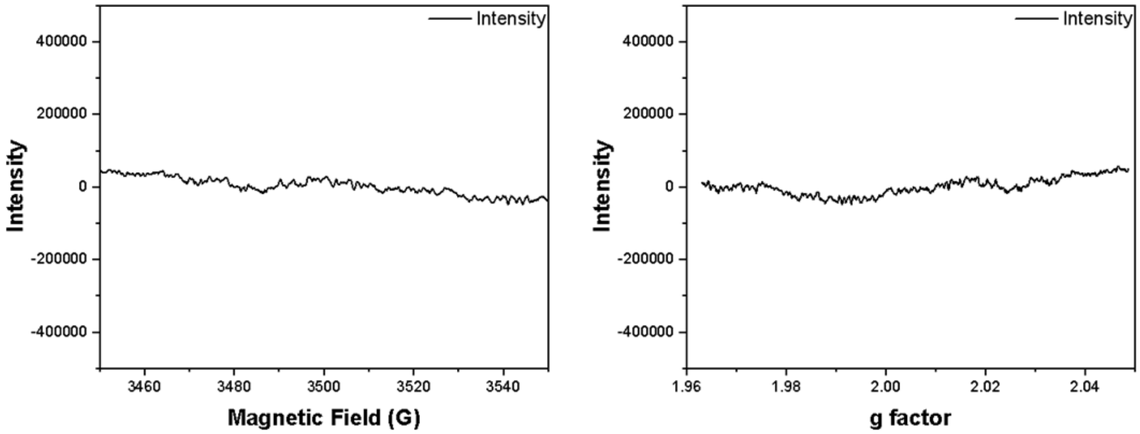

Figure S35. EPR spectra of METO-1+MMA.

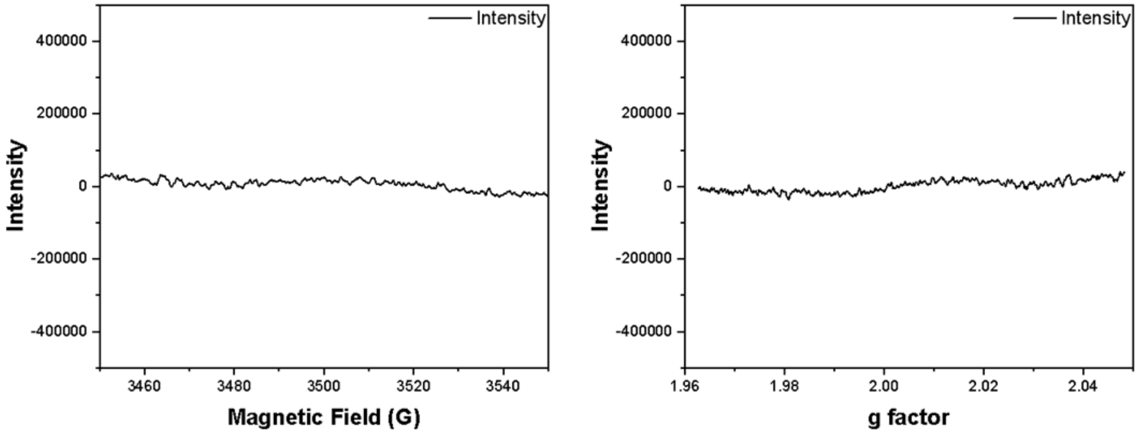

Figure S36. EPR spectra of METO-3+MMA.

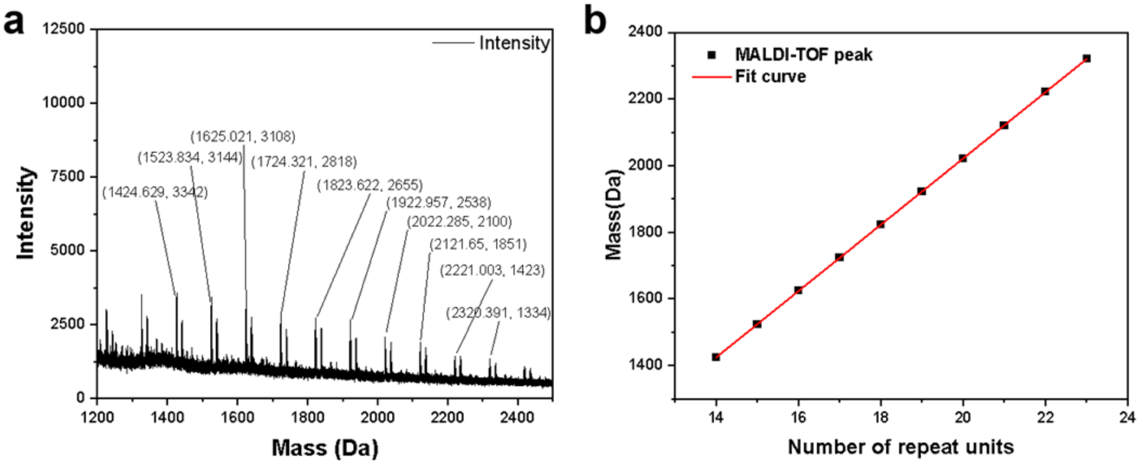

Figure S37. (a) MALDI-TOF-MS spectrum of a PMMA sample. (b) M/z of some peaks and the fit curve.

We selected a sample with a relatively low average molecular weight and analyzed it using MALDI-TOF-MS, discovering a clear pattern of missing repeat unit peaks. By fixing the intercept at 23 (Na^+) for fitting, we obtained a slope of 99.96, which is close to the relative molecular mass of MMA (100.12). By fixing the slope at 100.12, we obtained an intercept of 20.10, which is close to the relative atomic mass of the Na^+ ion (22.98).

^{1}H NMR spectra of polymers and method for determining conversion:

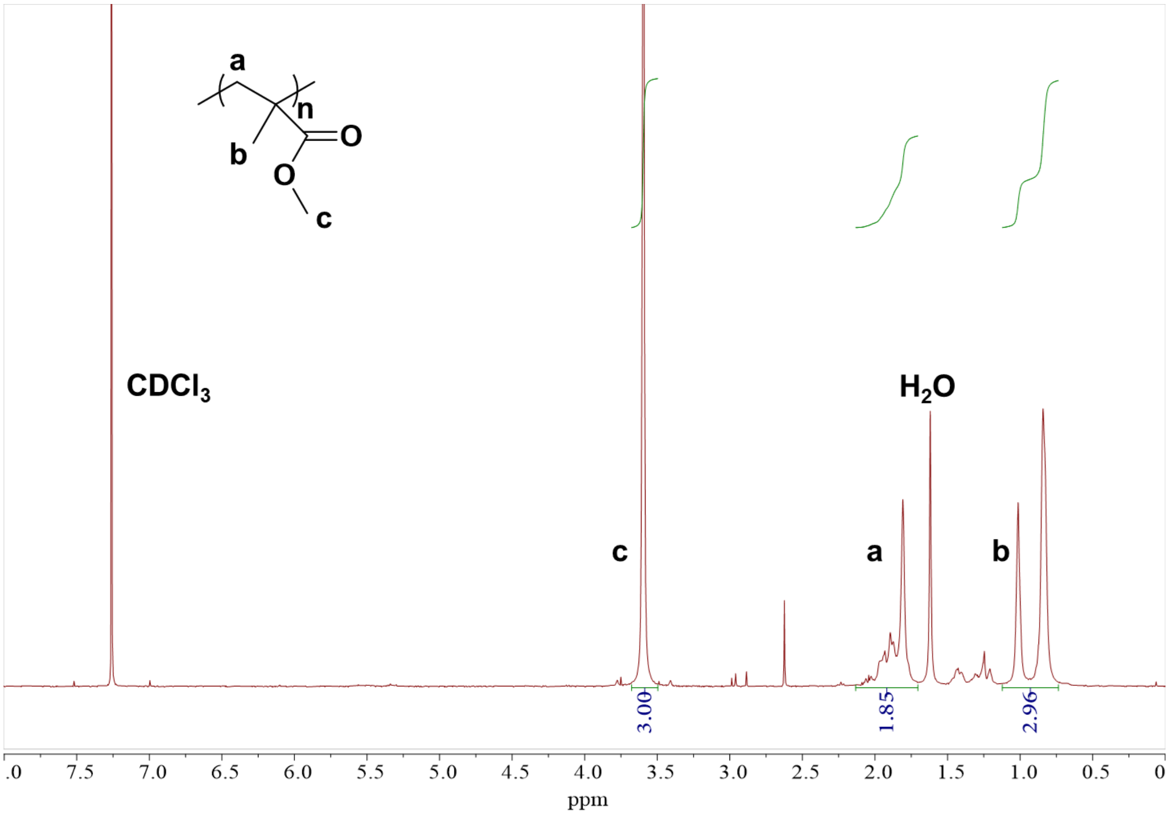

Figure S38.^{1}H NMR spectrum of PMMA. ($CDCl_3$)

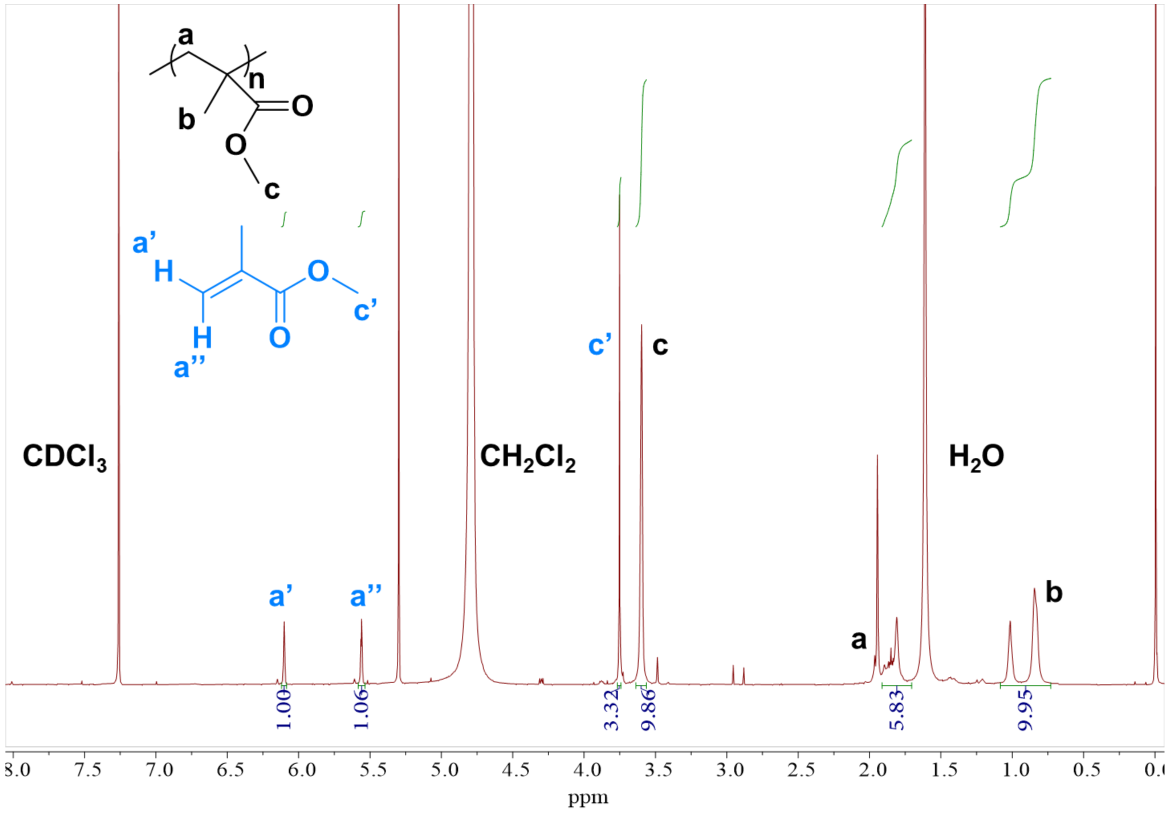

Figure S39.[1]**H NMR spectrum of the mixture of MMA and PMMA after polymerization. ($CDCl_3$)**

Calculation of the conversion: $k = \frac{c}{c'}$ $\alpha(\text{conversion}) = \frac{k}{k+1}$

Example: k = 2.97 α = 74.81%

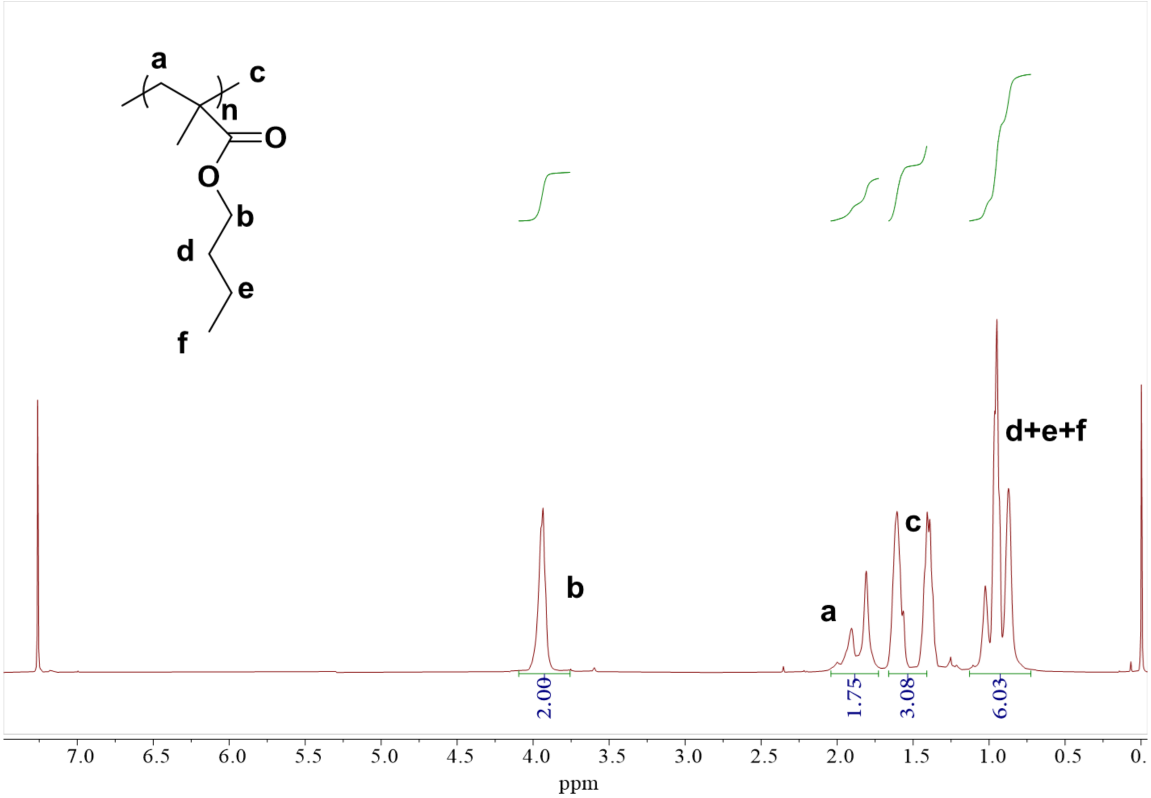

Figure S40.^{1}H NMR spectrum of PBMA. ($CDCl_3$)

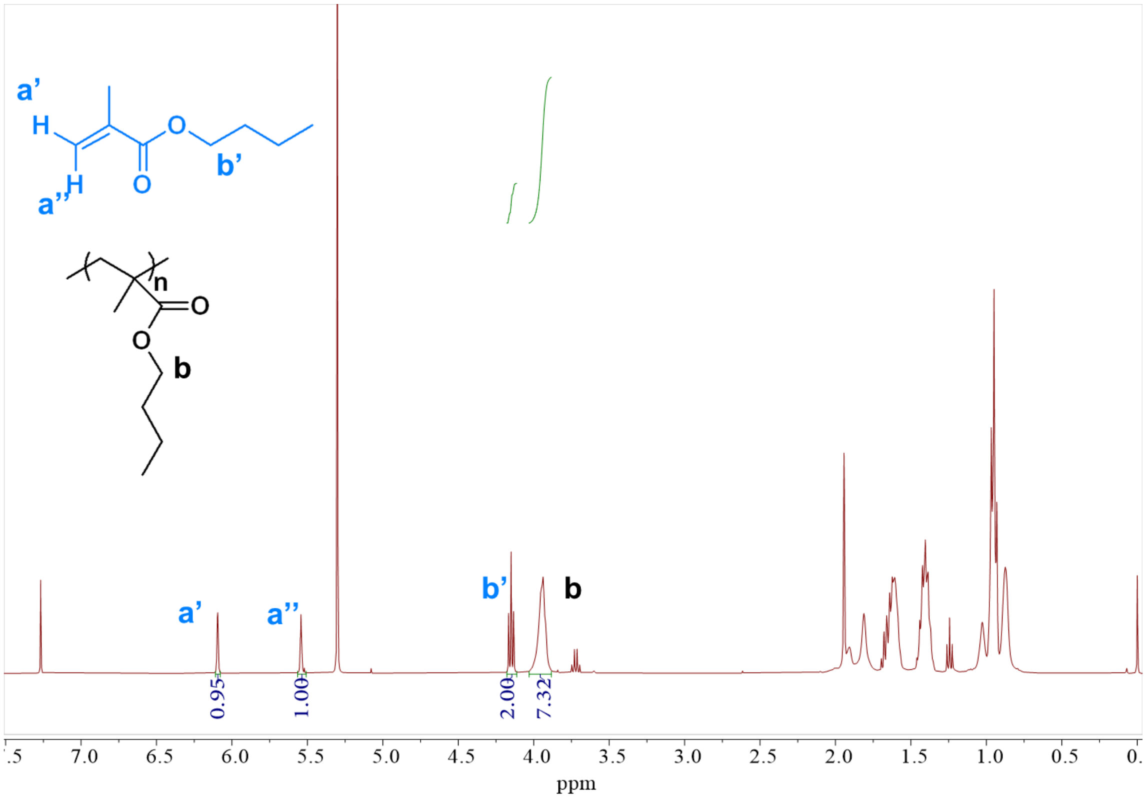

Figure S41. ^{1}**H NMR spectrum of the mixture of BMA and PBMA after polymerization. ($CDCl_3$)**

Calculation of the conversion: $k = \frac{b}{b'}$ $\alpha(\text{conversion}) = \frac{k}{k+1}$

Example: k = 3.66 α =78.54 %

Photocatalysis of another well-studied triplet-sensitized photochemical reaction:

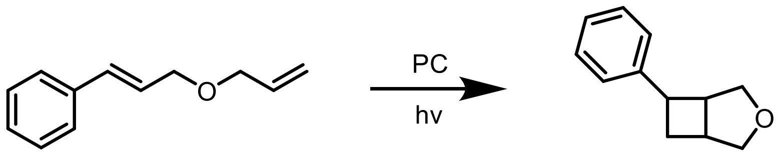

d1 d2

Figure S42. Photochemical reaction of d1.

Dissolve d1 (0.35 g, 2 mmol) in 2 mL of acetonitrile (1 M), add 0.02 mmol of photocatalyst, and react under 390 nm light irradiation for 6 hours. Concentrate the reaction mixture and characterize the yield by ^{1}H NMR.

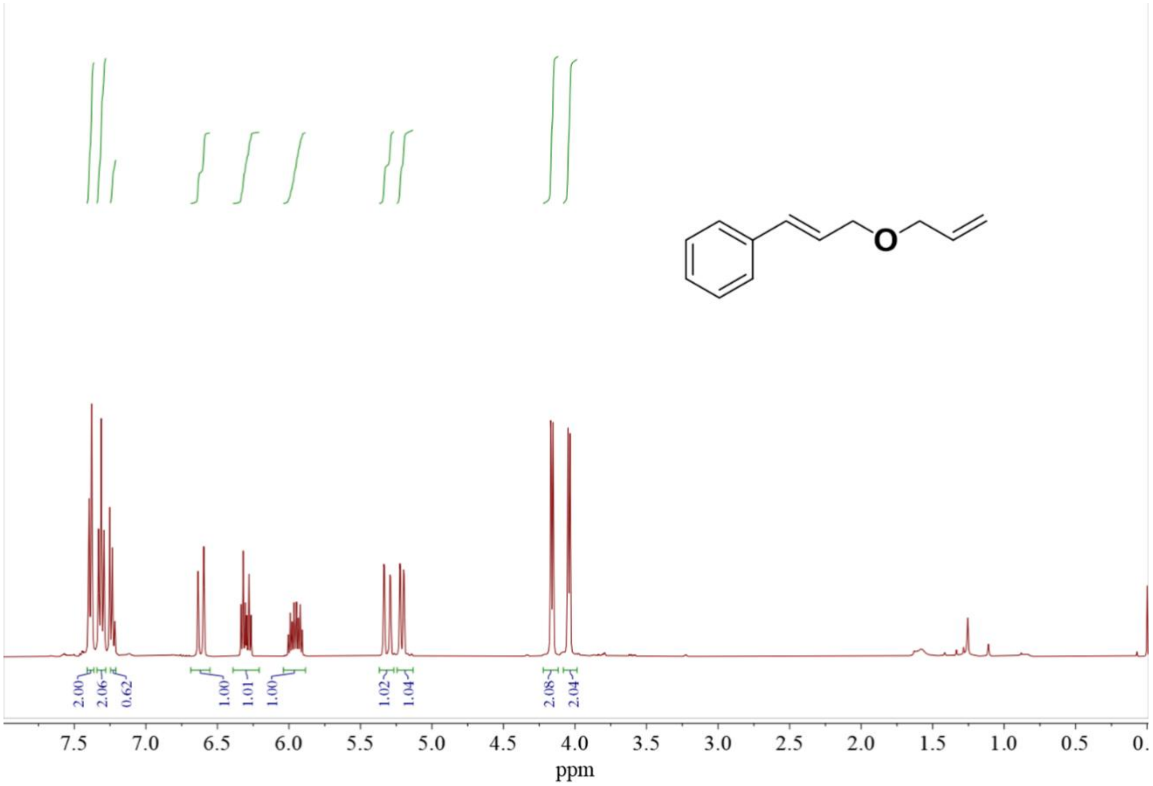

Figure S43.[1]**H NMR spectrum of d1. ($CDCl_3$)**

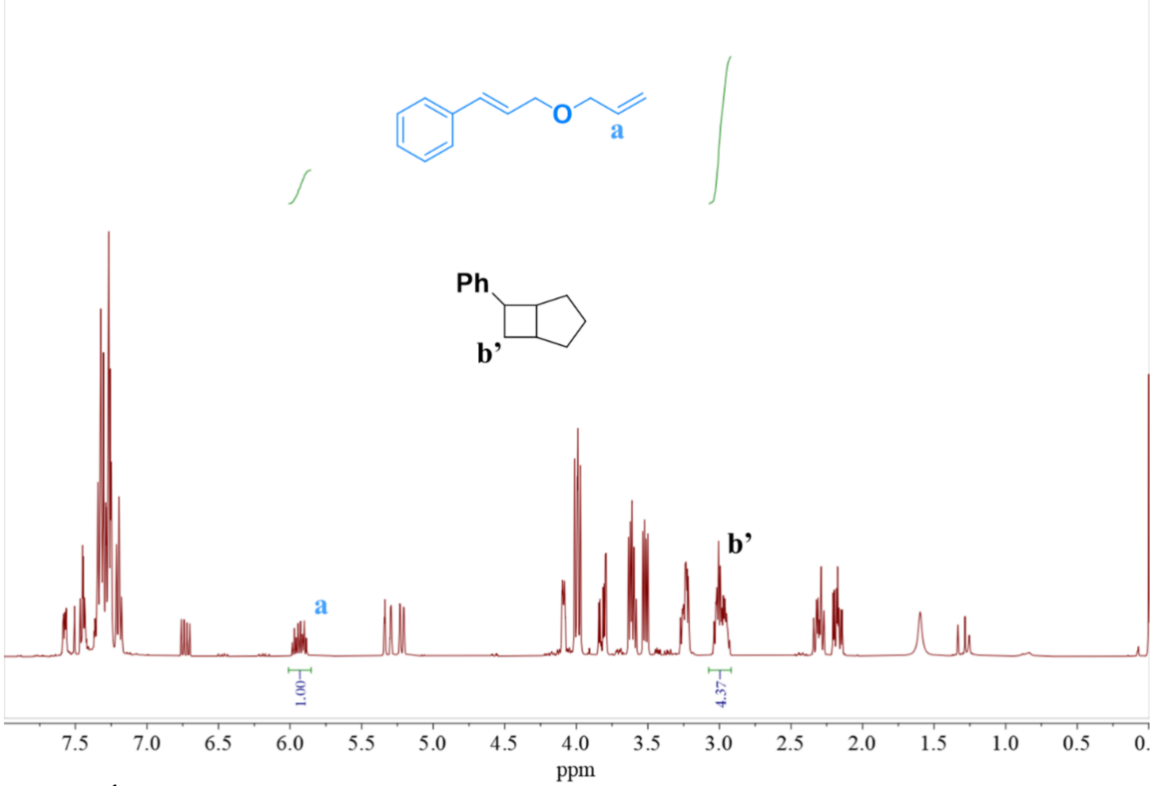

Figure S44.[1]**H NMR spectrum of mixture after the photo-induced cycloaddition. ($CDCl_3$)**

Calculation of the conversion: $k = \frac{b'}{2a}$ $\alpha(\text{conversion}) = \frac{k}{k+1}$

Example: k = 2.18 α = 68.85%

The conversion rate of METO-3 as a photocatalyst is 68.9%, and the conversion rate of METO-4 as a photocatalyst is 45.2%.

Synthesis of d4 and d5

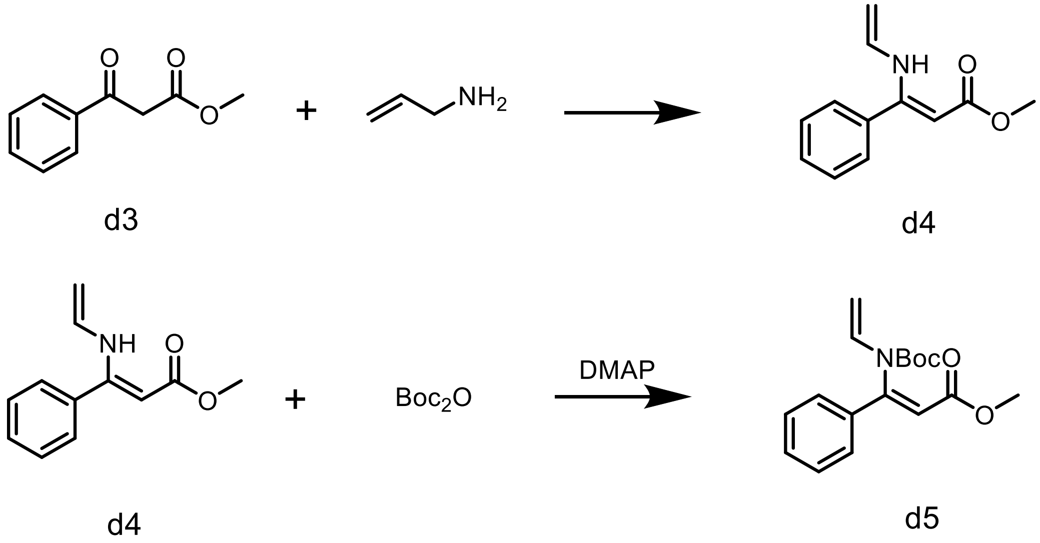

Figure S45. Synthesis of d4 and d5.

Acetic acid (3.36 g, 56 mmol) was added to methyl 3-oxo-3-phenylpropanoate (5.0 g, 28 mmol), triethylamine (5.66 g, 56 mmol) and 3-aminopropene hydrochloride (5.2 mL, 56 mmol) in EtOH (80 mL). The resulting solution was stirred at reflux overnight. The reaction mixture was evaporated and the crude product was purified by flash silica chromatography, elution gradient 5 to 30% EtOAc in petroleum ether. Pure fractions were evaporated to dryness to afford methyl (E)-3-(allylamino)-3-phenylacrylate (d4, 3.50 g, 62 %) as a colourless oil.

^{1}H NMR (400 MHz, $CDCl_3$) δ 8.61 (s, 1H), 7.40 – 7.31 (m, 5H), 5.78 (ddt, *J* = 17.1, 10.1, 4.9 Hz, 1H), 5.21 (dq, *J* = 17.2, 1.7 Hz, 1H), 5.11 (dq, *J* = 10.3, 1.6 Hz, 1H), 4.64 (s, 1H), 3.73 – 3.65 (m, 5H).

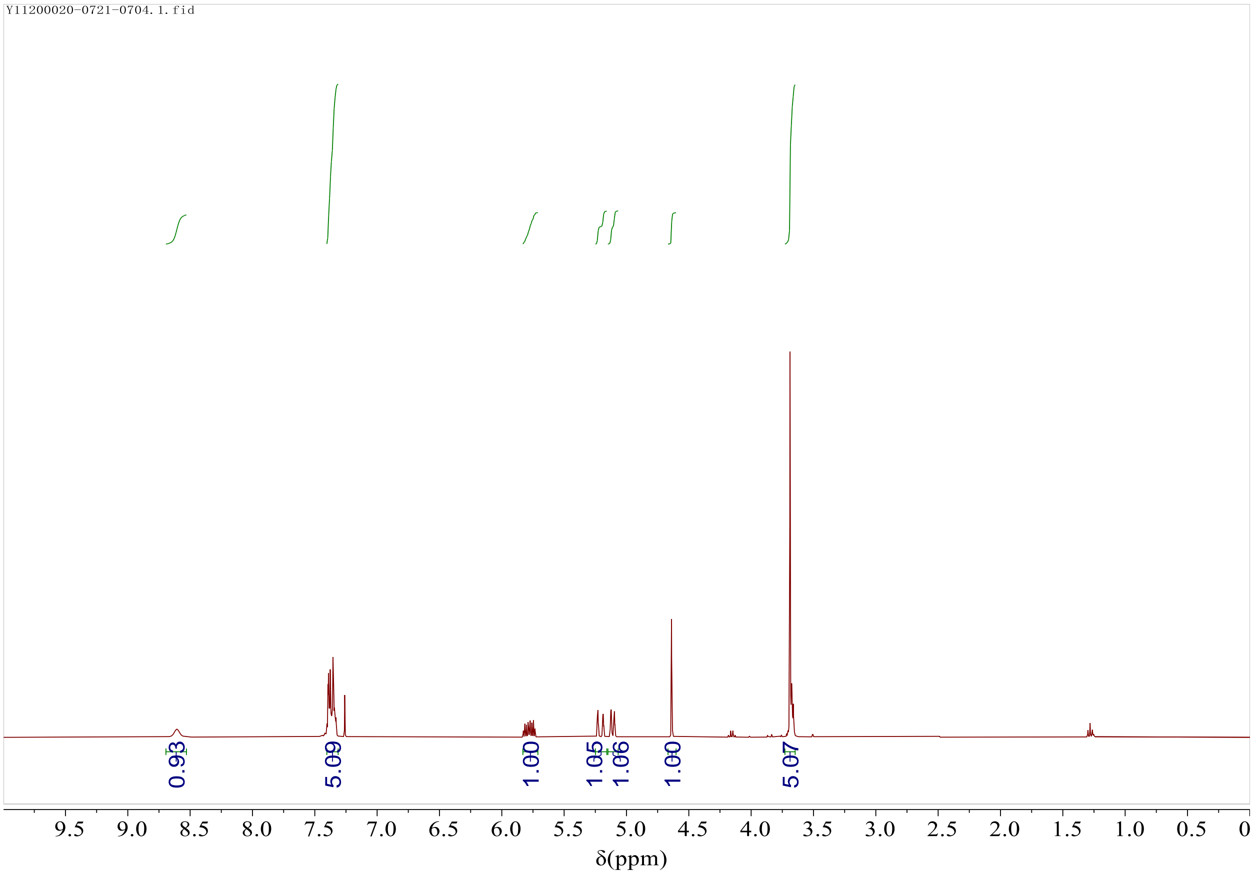

Figure S46. **^{1}H NMR spectrum of d4. ($CDCl_3$)**

DMAP (0.061 g, 0.5 mmol) was added to methyl (*E*)-3-(allylamino)-3-phenylacrylate (2.17 g, 10 mmol) and di-tert-butyl dicarbonate (2.18 g, 15 mmol) in acetonitrile (25 ml). The resulting solution was stirred at 65 ℃ overnight. The reaction mixture was evaporated, the crude product was purified by flash silica chromatography, elution gradient 0 to 40% EtOAc in petroleum ether. Pure fractions were evaporated to dryness to afford d5 (1.24 g, 41%) as a pale yellow oil.

^{1}H NMR (400 MHz, $CDCl_3$) δ 7.38 – 7.31 (m, 5H), 5.96 – 5.89 (m, 1H), 5.85 (s, 1H), 5.24 – 5.16 (m, 2H), 4.24 (dt, *J* = 5.2, 1.6 Hz, 2H), 3.57 (s, 3H), 1.14 (s, 9H).

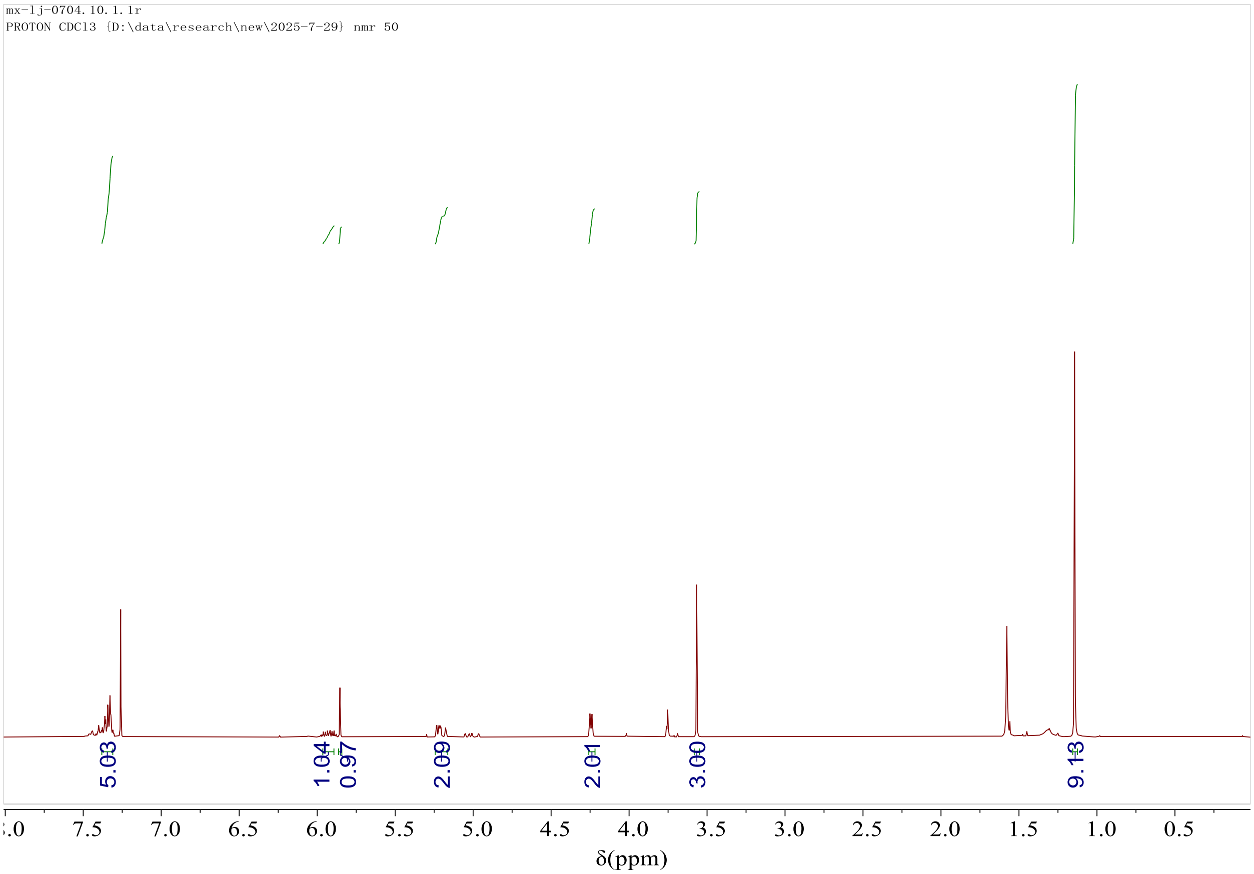

Figure S47.^{1}H NMR spectrum of d5. (CDCl₃)

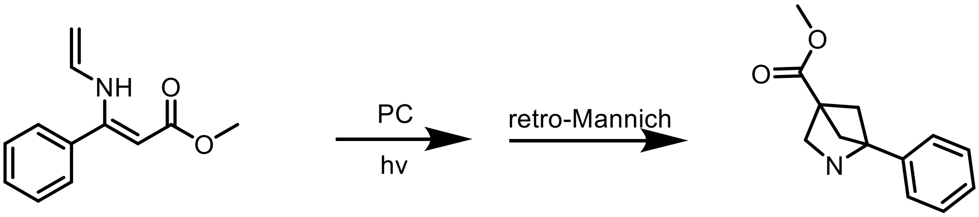

d4 d6

Figure S48. Photochemical reaction of d4.

Dissolve d4 (0.41 g, 2 mmol) in 2 mL of acetonitrile (1 M), add 0.02 mmol of photocatalyst, and react under 390 nm light irradiation for 12 hours. Concentrate the reaction mixture and characterize the yield by ^{1}H NMR. Attempted to use flash silica chromatography to separate the product, but the yield was too low to obtain the product.

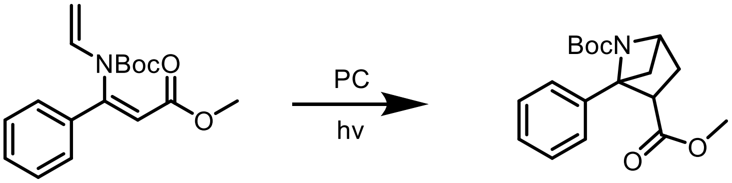

d5 d7

Figure S49. Photochemical reaction of d5.

Dissolve d4 (0.31 g, 1 mmol) in 1 mL of acetonitrile (1 M), add 0.01 mmol of photocatalyst, and react under 390 nm light irradiation for 12 hours. Concentrate the reaction mixture and characterize the yield by ^{1}H NMR. After the reaction, the solution was evaporated and then frozen at -20 ℃. It was washed with refrigerated hexane and thawed under vacuum to room temperature, yielding a pale yellow gel-like solid.

^{1}H NMR (400 MHz, Chloroform-*d*) δ 7.41 (d, *J* = 7.5 Hz, 2H), 7.31 (t, *J* = 7.1 Hz, 2H), 7.27 – 7.23 (m, 1H), 3.94 – 3.90 (m, 1H), 3.70 (s, 3H), 3.58 – 3.54 (m, 1H), 3.06 (dd, *J* = 7.2, 3.0 Hz, 2H), 1.93 – 1.85 (m, 2H), 1.07 (s, 9H).

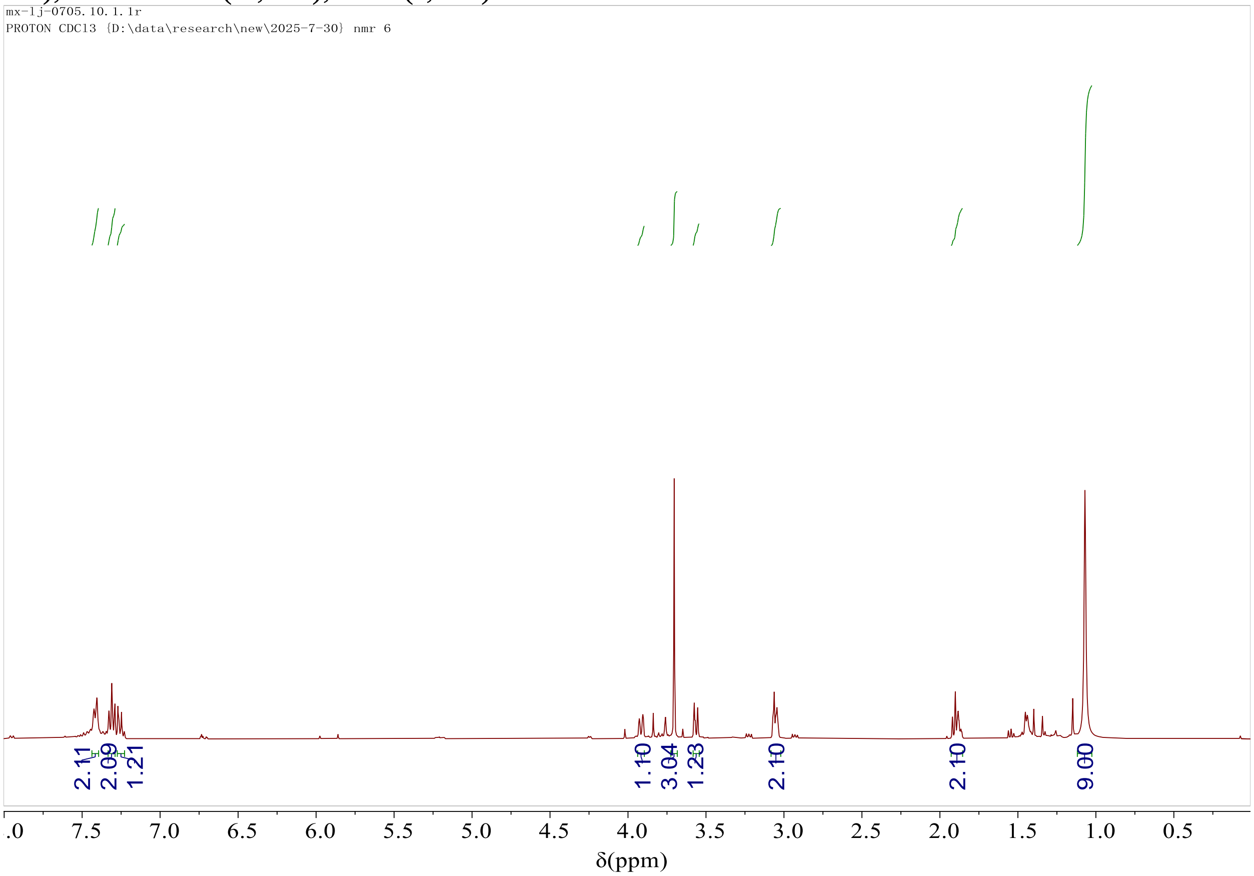

Figure S50.^{1}H NMR spectrum of d7. ($CDCl_3$)

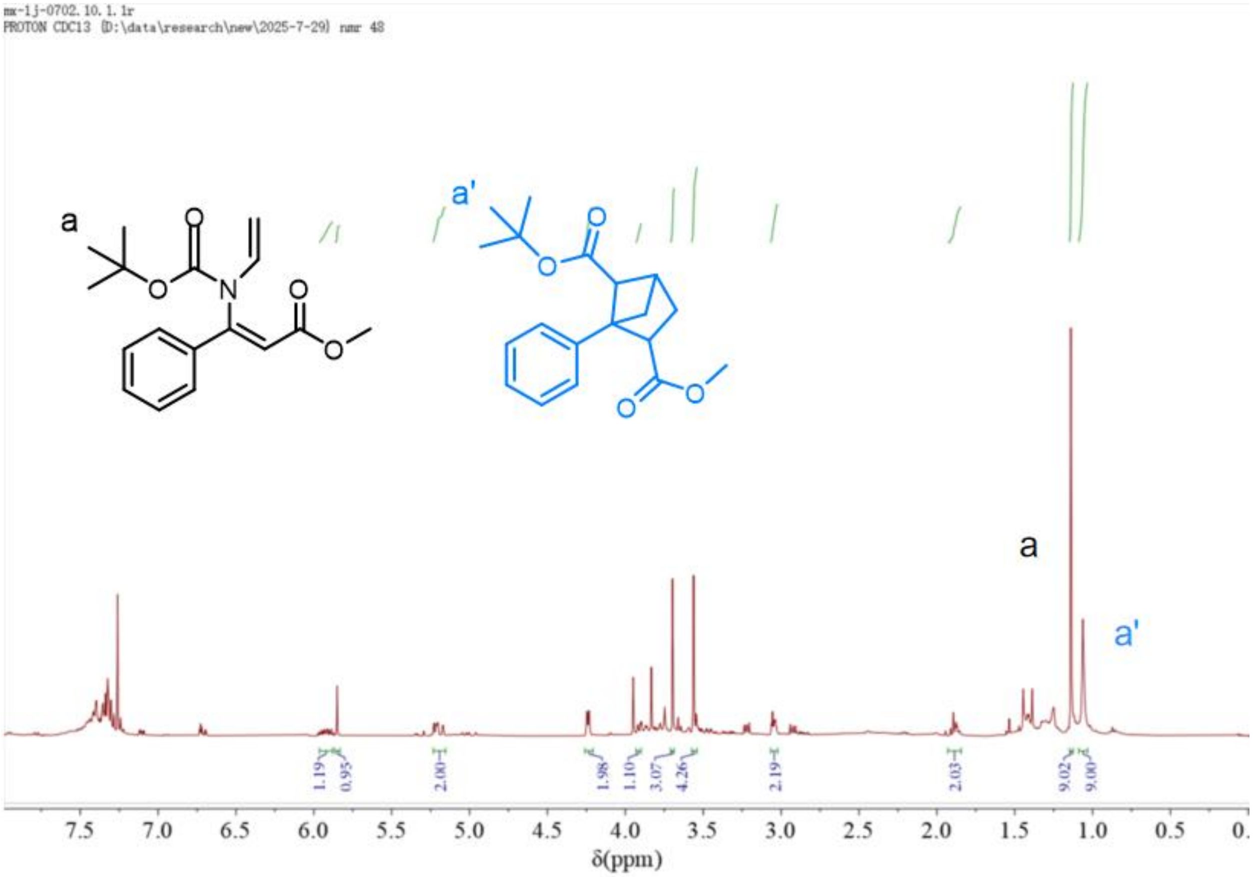

Figure S51. ^{1}H NMR spectrum of mixture after the photo-induced cycloaddition of d5. ($CDCl_3$) Calculation of the conversion: $k = \frac{a'}{a}$ $\alpha(\text{conversion}) = \frac{k}{k+1}$

Example: k = 0.998 α = 49.94%

The conversion rate of METO-3 as a photocatalyst is 85.2%, and the conversion rate of METO-4 as a photocatalyst is 49.9%.

Time-dependent density functional theory (TD-DFT) calculations were carried out on the Gaussian G09 program at the M062x/def2tzvpp // M062x/def2svp set. The T_1 state energy of the monomer and PC were calculated as follows:

Table S11. The T_1 energy of PCs and monomers mentioned in this article.

	**T_1 energy (eV)**	**Relative wavelength (nm)**
**MA**	**2.75**	450.9
**MMA**	**2.63**	471.5
**BMA**	**2.69**	461.0
**St**	**2.54**	488.2
**PEGMA(n=8)**	**2.61**	475.1
**METO-1**	**2.71**	457.6
**METO-2**	**2.89**	429.1
**METO-3**	**3.26**	380.4
**METO-4**	**3.15**	393.7
**CBDAC**	**2.34**	529.9
**4Cz2CN**	**2.62**	473.3
**DPA**	**1.94**	639.2
**PDI-C_8**	**1.48**	837.8

Light source data mentioned in this article:

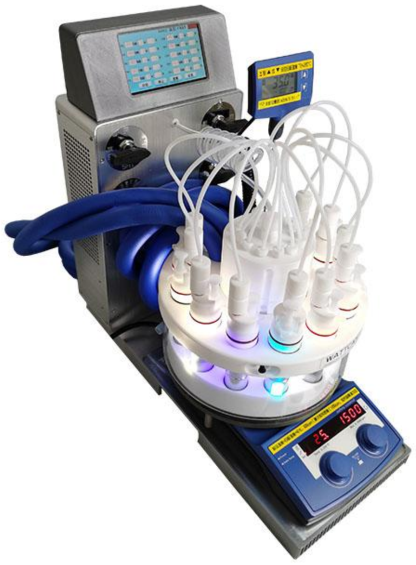

Figure S52. The parallel light reactor used in this paper.

All light reactions (except those in sunlight) were carried out on WATTECS WP-TEC-1020 parallel reactor. Light sources for the photoreactor are provided by Shenzhen Learnew optoelectronics technology co ltd. Uninterrupted water flow into the jacket during the whole reaction process to keep cooling and ensure that the temperature of the reaction system remains below 25℃.

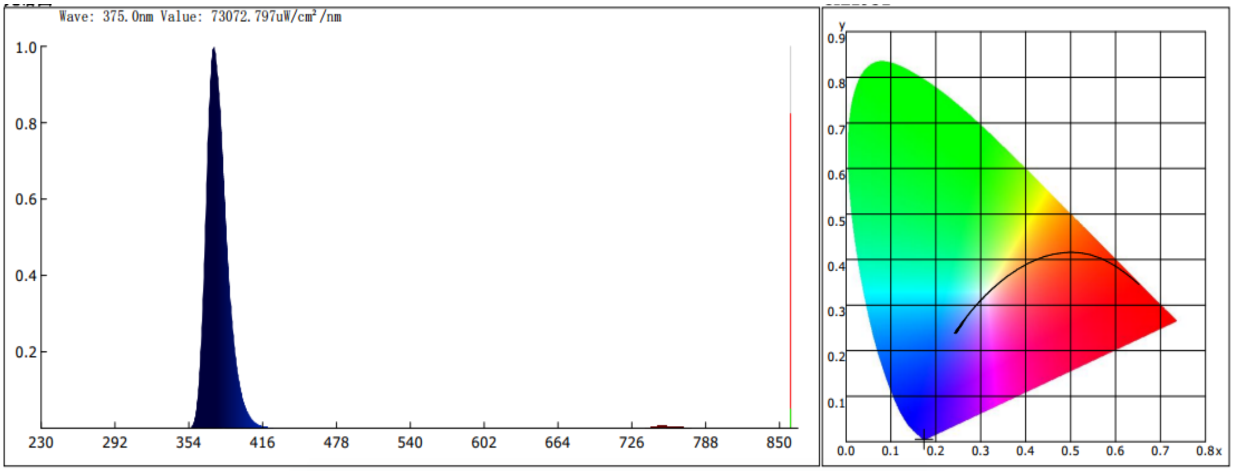

Figure S53. Emission spectrum of 365nm light source.

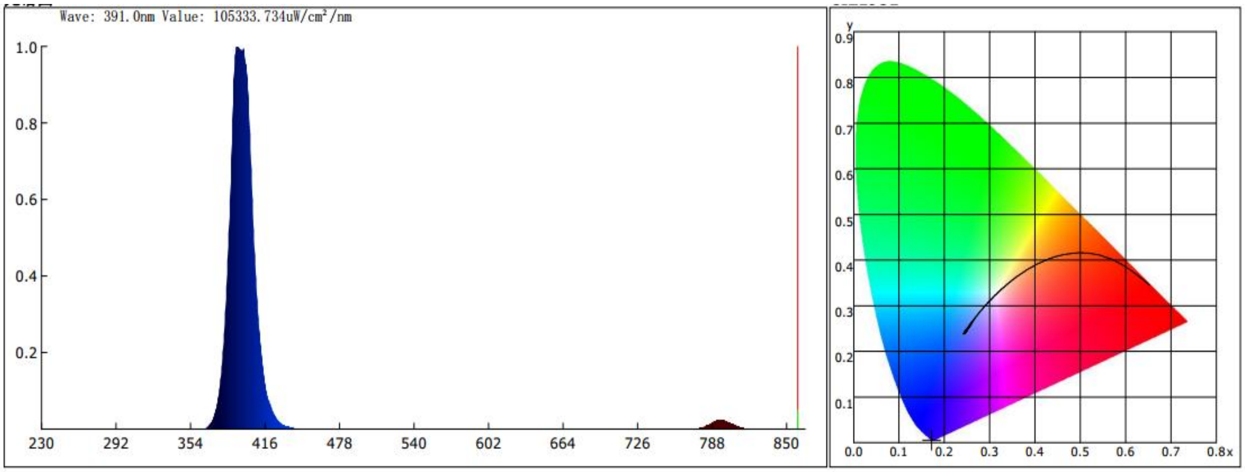

Figure S54. Emission spectrum of 390nm light source.

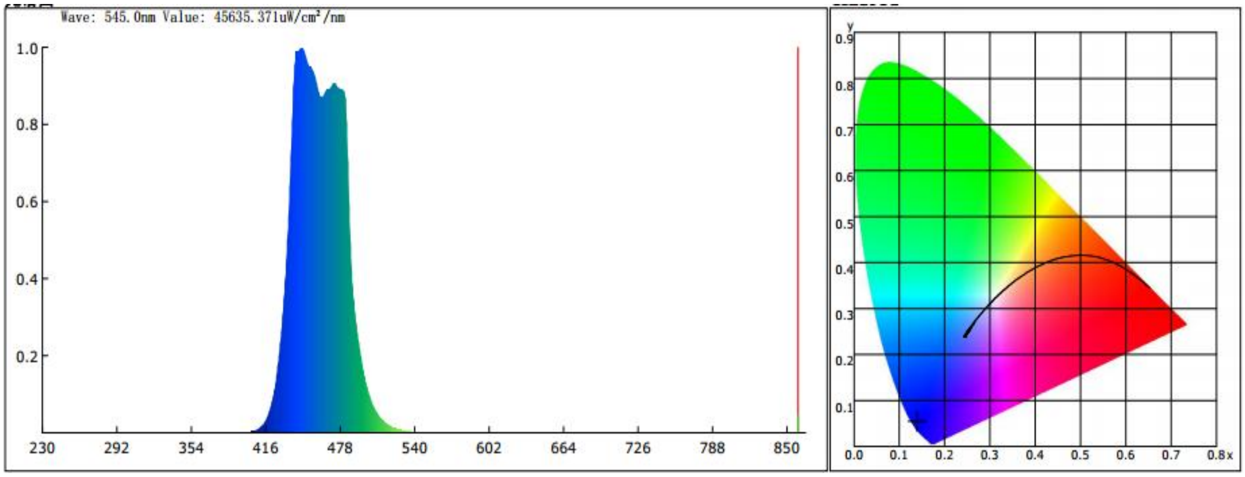

Figure S55. Emission spectrum of 460nm light source.

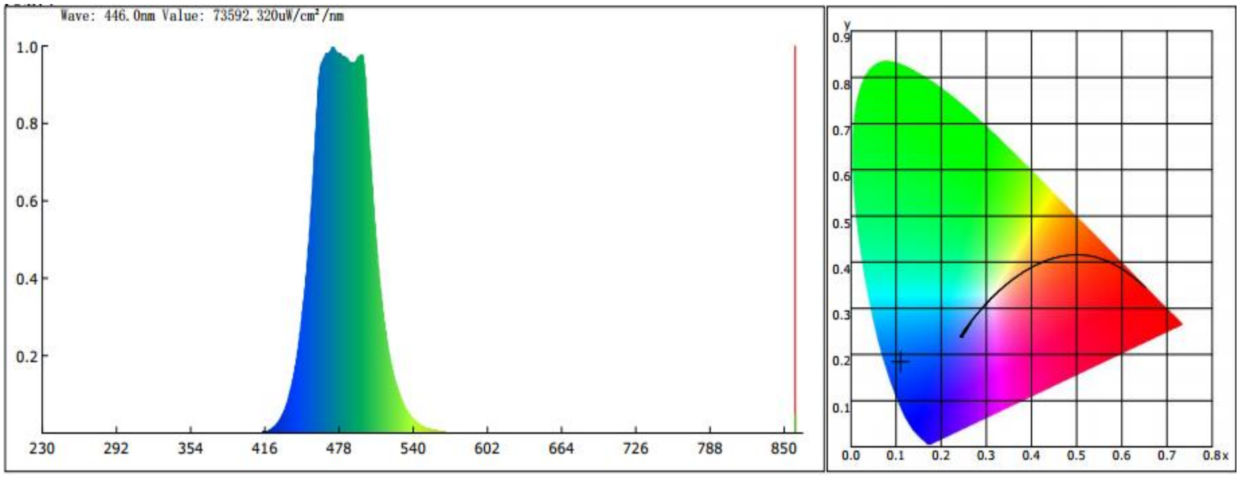

Figure S56. Emission spectrum of 480nm light source.

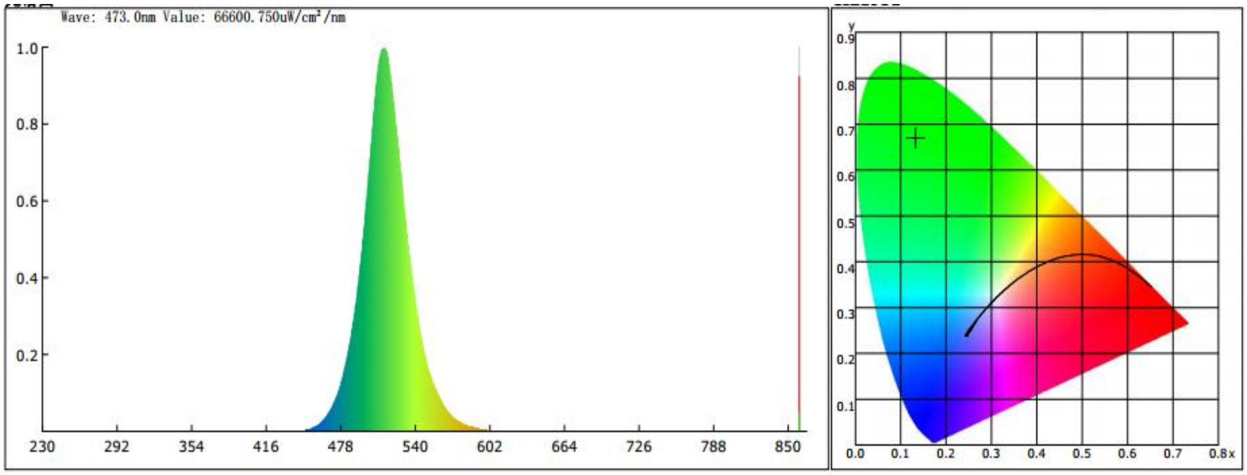

Figure S57. Emission spectrum of 510nm light source.

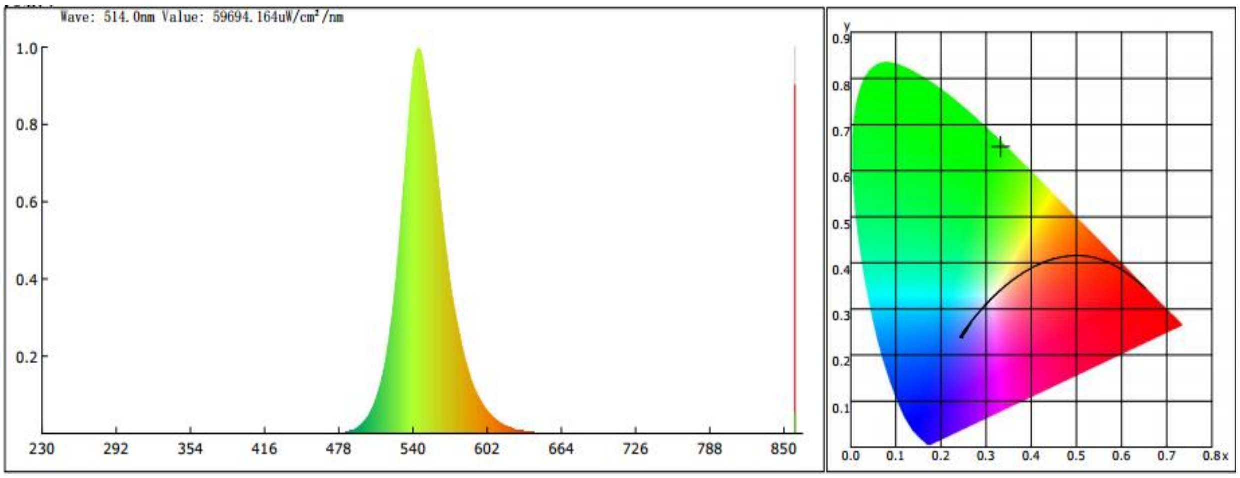

Figure S58. Emission spectrum of 550nm light source.

References

1 Qu G., Jiang T., Liu T. *et al.* Temperature-Responsive Ratiometric Au Nanoclusters (NCs) Fabricated through α-Cyclodextrin-Protected Au NCs and Au@GSH NCs. *Ind Eng Chem Res* 2023; **62**, 7365-72.

2 Ma L., Sun S., Ding B. *et al.* Highly Efficient Room-Temperature Phosphorescence Based on Single-Benzene Structure Molecules and Photoactivated Luminescence with Afterglow. *Adv Funct Mater* 2021; **31**, 2010659.

3 Xue W., Zhang X., Zhu W. *et al.* Large-scale heterogeneous synthesis of monodisperse high performance colloidal CsPbBr3 nanocrystals. *Fundam Res* 2022.

4 Elliott L. D., Kayal S., George M. W. *et al.* Rational Design of Triplet Sensitizers for the Transfer of Excited State Photochemistry from UV to Visible. *J Am Chem Soc* 2020; **142**, 14947-56.

5 Pu C., Zhou J., Lai R. *et al.* Highly reactive, flexible yet green Se precursor for metal selenide nanocrystals: Se-octadecene suspension (Se-SUS). *Nano Res* 2013; **6**, 652-70.

6 Bae W. K., Padilha L. A., Park Y.-S. *et al.* Controlled Alloying of the Core–Shell Interface in CdSe/CdS Quantum Dots for Suppression of Auger Recombination. *ACS Nano* 2013; **7**, 3411-9.
